# Effects of high-molecular-weight hyaluronic acid on inflammatory and matrix-associated responses in a chondrocyte–osteoclast co-culture model

**DOI:** 10.1186/s13018-026-07024-7

**Published:** 2026-07-13

**Authors:** Adam Eid, Kavitha Raja, Dineshwary Suresh, Surendra Singh Rawat, Ivan James Prithishkumar, Thomas Nau, Nerissa Naidoo

**Affiliations:** 1 College of Medicine, Mohammed Bin Rashid University of Medicine and Health Sciences, Dubai Health, Building 14, P.O. Box 505055, Dubai, United Arab Emirates; 2 Faculty of Medicine, Health and Life Science, Swansea University, Wales, UK; 3 King’s College Hospital London Dubai, Dubai, United Arab Emirates

**Keywords:** Osteoarthritis, Hyaluronic acid, Chondrocytes, Osteoclasts, NF-κB signaling, CD44–PI3K/Akt, Inflammation, Extracellular matrix

## Abstract

**Background:**

Osteoarthritis (OA) is a degenerative joint disorder marked by chronic inflammation, extracellular matrix (ECM) breakdown, and subchondral bone remodeling, ultimately leading to loss of cartilage integrity and joint function. Hyaluronic acid (HA), a key component of synovial fluid, contributes to joint lubrication and has been implicated in the regulation of inflammatory and anabolic processes. High-molecular-weight (HMW) HA (> 1 MDa) is reported to exert anti-inflammatory and matrix-preserving effects; however, its role in multicellular systems remains incompletely understood. In this study, we investigated the effects of HMW HA using an in vitro chondrocyte–osteoclast co-culture model designed to reflect key features of the osteoarthritic microenvironment.

**Methods:**

Bone marrow-derived mesenchymal stem cells were differentiated into chondrocytes, while RAW 264.7 macrophages were induced into osteoclast-like cells using M-CSF and RANKL. Cells were co-cultured at a 1:1 ratio and stimulated with lipopolysaccharide (LPS; 1 µg/mL) to induce inflammatory stress, followed by HA treatment (50–500 µg/mL) for 24–48 h. Cell viability, morphology, and TRAP staining were assessed. Expression of inflammatory and matrix-associated markers was analysed by Western blot, qPCR, and ELISA. Cell-cycle distribution and DNA integrity were evaluated using flow cytometry and agarose gel electrophoresis.

**Results:**

LPS exposure reduced metabolic activity, altered morphology, and was associated with increased expression of NF-κB-related inflammatory markers and elevated expression of IL-1β, TNF-α, MMP-13, and ADAMTS-5, alongside reduced COL2A1 and ACAN levels. HA treatment improved metabolic activity and preserved cellular morphology in a concentration-dependent manner, with the most pronounced effects observed at 200–500 µg/mL. HA-treated cultures showed lower levels of inflammatory and catabolic markers together with increased expression of ECM-associated genes. In addition, HA treatment was associated with a more balanced cell-cycle profile and reduced DNA fragmentation.

**Conclusion:**

HMW HA was associated with coordinated modulation of inflammatory and matrix-related markers in an LPS-induced co-culture system. These findings suggest that HA may contribute to maintaining cellular homeostasis under inflammatory conditions in vitro. Further studies are needed to clarify the underlying mechanisms and translational relevance.

**Supplementary Information:**

The online version contains supplementary material available at https://doi.org/10.1186/s13018-026-07024-7.

## Introduction

Osteoarthritis (OA) is a chronic degenerative joint disorder characterized by the progressive deterioration of articular cartilage, alterations in subchondral bone architecture, and dysregulation of synovial tissue homeostasis [1, 2]. With a global prevalence exceeding 500 million individuals, OA represents a leading cause of chronic pain, reduced mobility, and long-term disability [3, 4]. At the cellular level, disease progression is driven by a loss of chondrocyte homeostasis, wherein catabolic activity overwhelms intrinsic repair mechanisms, resulting in extracellular matrix (ECM) degradation, sustained inflammation, and compromised joint function [5].

Pro-inflammatory cytokines, particularly interleukin-1β (IL-1β) and tumor necrosis factor-α (TNF-α), play central roles in OA pathogenesis through activation of the nuclear factor-κB (NF-κB) signaling pathway. NF-κB activation induces the expression of matrix-degrading enzymes such as matrix metalloproteinase-13 (MMP-13) and a disintegrin and metalloproteinase with thrombospondin motifs-5 (ADAMTS-5), which target type II collagen and aggrecan key structural components of cartilage ECM [6, 7]. In parallel, inflammatory crosstalk between chondrocytes and osteoclast progenitors contributes to aberrant subchondral bone remodeling, further amplifying inflammatory signaling within the osteoarthritic joint microenvironment [8, 9].

Current pharmacological interventions, including non-steroidal anti-inflammatory drugs and intra-articular corticosteroids, are largely limited to symptomatic management and fail to modify disease progression or restore joint structure [10]. This therapeutic gap has prompted increasing interest in biologically active macromolecules and biomaterial-based strategies capable of modulating inflammatory signaling while supporting cartilage matrix preservation.

Hyaluronic acid (HA) is an endogenous high-molecular-weight glycosaminoglycan (> 1 MDa) and a major constituent of synovial fluid, where it contributes to joint lubrication, viscoelasticity, and mechanical load distribution [11, 12]. Beyond its biophysical functions, HA actively regulates cellular behaviour through interactions with surface receptors such as CD44 and Toll-like receptor 4 (TLR4), thereby influencing inflammatory responses, oxidative stress, and anabolic signalling pathways [13]. Importantly, the biological activity of HA is molecular-weight dependent: high-molecular-weight (HMW) HA exhibits anti-inflammatory and anti-catabolic properties, whereas fragmented or low-molecular-weight HA has been associated with pro-inflammatory signalling [14, 15]. Experimental and clinical studies have reported that exogenous HMW HA can suppress NF-κB activation, reduce IL-1β and TNF-α production, and enhance the synthesis of cartilage-specific ECM components, including type II collagen and aggrecan [16, 17]. Previous studies have also demonstrated that high-molecular-weight hyaluronic acid modulates inflammatory responses in both chondrocytes and macrophage-derived cells [18–20]. Despite these observations, the mechanisms by which HA modulates inflammatory interactions between chondrocytes and osteoclasts remain insufficiently defined. Therefore, this study aimed to investigate the effects of high-molecular-weight HA on inflammatory and matrix-associated markers in an LPS-induced chondrocyte–osteoclast co-culture model that mimics key aspects of the osteoarthritic microenvironment.

## Materials and methods

### Study design

This study was conducted as a controlled in vitro investigation to assess the effects of hyaluronic acid (HA) on inflammatory signalling, osteoclastogenesis, and extracellular matrix (ECM) regulation in a chondrocyte–osteoclast co-culture system. Lipopolysaccharide (LPS) was used to induce inflammatory stress, after which HA was administered at increasing concentrations to evaluate its effects on cell viability, differentiation status, and ECM-related molecular responses.

### Ethical and biosafety compliance

All experimental procedures were performed in compliance with institutional biosafety and ethical regulations approved by the Mohammed Bin Rashid University of Medicine and Health Sciences (MBRU), Dubai, UAE. Cell culture work was conducted in accordance with MBRU Institutional Biosafety Committee (IBC) guidelines and international Good Laboratory Practice (GLP) standards. All reagents and biological materials were sourced from certified commercial suppliers and used exclusively for research purposes.

### Preparation of hyaluronic acid stock solution

The hyaluronic acid used in this study was a commercially available high-molecular-weight formulation (Durolane^®^, Bioventus), consisting of stabilized, crosslinked hyaluronic acid with an approximate molecular weight range of 1–3 MDa. The gel was aseptically transferred from the pre-filled syringe into a sterile 15 mL conical tube inside a biosafety cabinet and equilibrated at room temperature for 30 min to reduce viscosity. The viscous HA was diluted with sterile phosphate-buffered saline (PBS; pH 7.2–7.4) at a 1:19 (v/v) ratio. The suspension was gently mixed by slow inversion until a homogeneous solution was obtained. All procedures were performed aseptically under laminar flow conditions, as the cross-linked nature of the gel prevented membrane filtration. The resulting suspension was designated as a 10 mg/mL stock working solution and aliquoted into sterile microtubes for short-term storage at 4 °C for up to three weeks.

For experimental treatments, the HA stock was freshly diluted in pre-warmed complete culture medium to obtain final concentrations of 50, 100, 200, and 500 µg/mL. The HA–medium mixture was pre-blended before addition to cell cultures to ensure even distribution.

### Cell culture and differentiation

Bone marrow–derived mesenchymal stem cells (BMSCs) used in this study were obtained as frozen cryovials from Cytion GmbH (Germany) and cultured in Dulbecco’s Modified Eagle Medium (DMEM; Gibco, Thermo Fisher Scientific, Waltham, MA, USA) supplemented with 10% fetal bovine serum (FBS; Gibco), 1% penicillin–streptomycin (100 U/mL and 100 µg/mL, respectively), and maintained at 37 °C in a humidified incubator with 5% CO₂. Upon reaching 80–90% confluence, cells were harvested using TrypLE Express (Gibco) and seeded for chondrogenic differentiation.

**Fig. 1:**
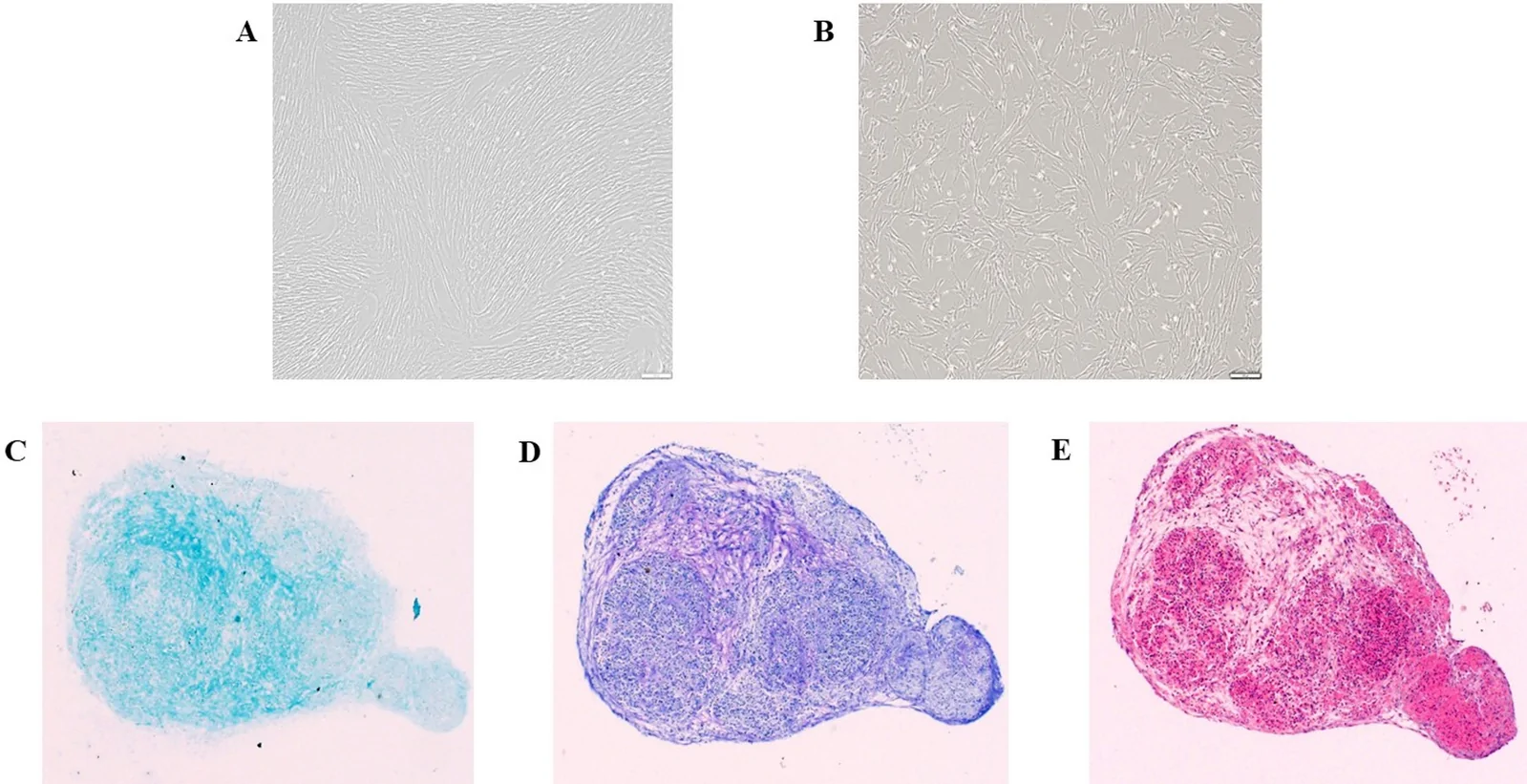
Morphological and histological characterization of BMSC-derived chondrocytes and spheroids. (**A**) Bone marrow–derived mesenchymal stem cells (MSCs) exhibited a typical spindle-shaped morphology under phase-contrast microscopy. (**B**) After 21 days of chondrogenic induction, differentiated chondrocytes displayed a polygonal, condensed morphology. (**C**) Alcian Blue staining of 3D spheroids showed blue coloration consistent with sulfated glycosaminoglycan deposition. (**D**) Toluidine Blue staining showed metachromatic regions indicating proteoglycan presence. (**E**) Hematoxylin and eosin (**H** &** E**) staining revealed cellular distribution within the spheroid structurer. Scale bars = 100 μm (**A–E**)

Chondrocyte differentiation was induced using chondrocyte differentiation medium (StemPro, Thermo Fisher Scientific) for 21 days, with medium changes every 48 h, following established MSC-to-chondrocyte differentiation protocols [18]. After differentiation, cells were maintained as three-dimensional spheroids in low-attachment culture plates to promote ECM deposition [19]. Differentiated chondrocytes exhibited positive Alcian Blue and Toluidine Blue staining, and the expression of cartilage-specific markers (COL2A1 and ACAN) supported chondrogenic differentiation. (Fig. 1). In addition, differentiation was further validated by assessing the expression of cartilage-specific markers, including COL2A1 and ACAN, at both mRNA and protein levels.

**Fig. 2:**
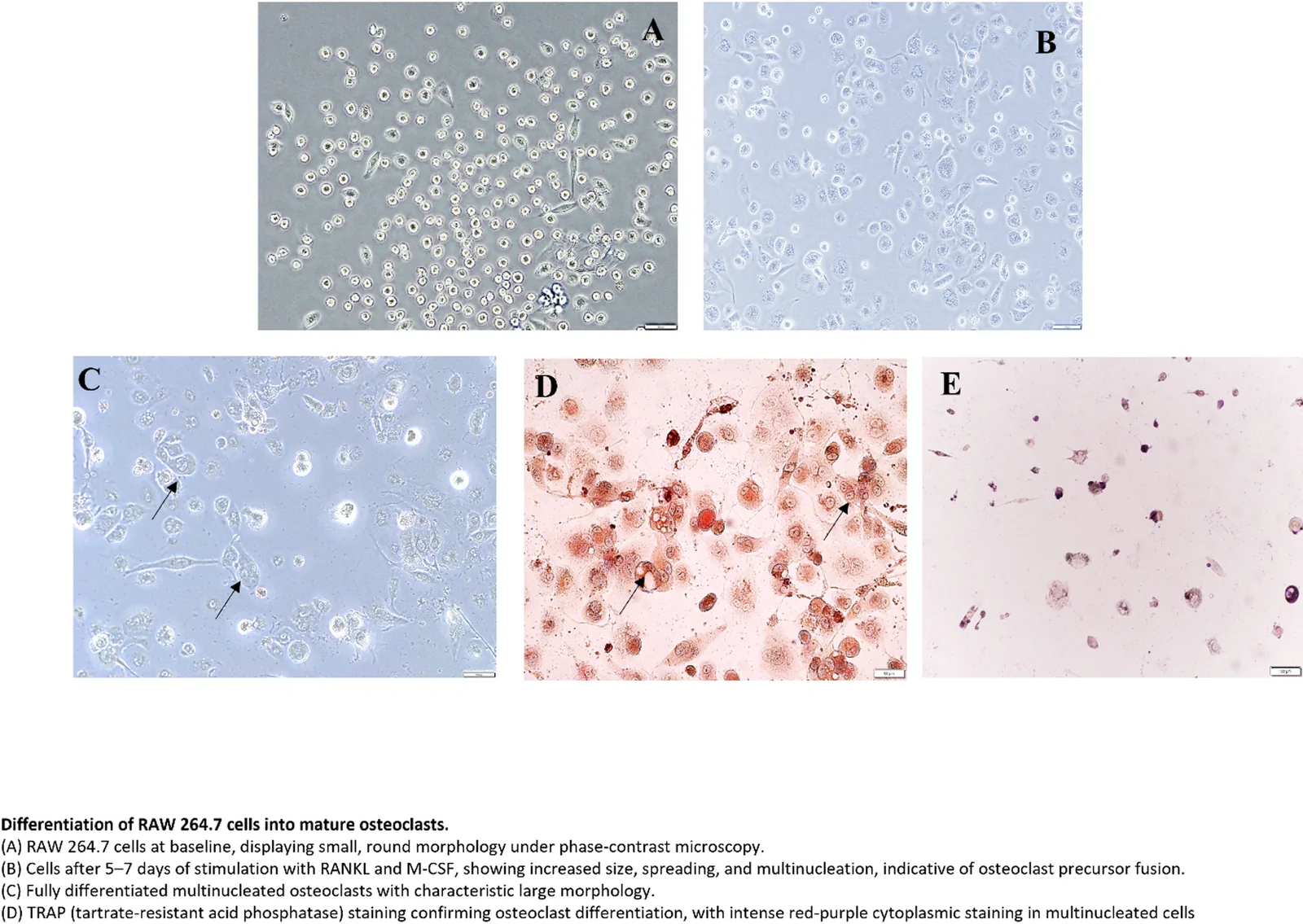
Osteoclast differentiation of RAW 264.7 cells. (**A**) RAW 264.7 cells at baseline, displaying small, round morphology under phase-contrast microscopy. (**B**) Cells after 5–7 days of stimulation with RANKL and M-CSF, showing increased cell size and spreading, consistent with early differentiation. (**C**) Cells exhibiting multinucleation and enlarged morphology consistent with osteoclast differentiation. (**D**) TRAP (tartrate-resistant acid phosphatase) staining demonstrating TRAP-positive osteoclast-like cells, with intense cytoplasmic staining in multinucleated cells. (**E**) Undifferentiated RAW 264.7 cells cultured without RANKL and M-CSF and subjected to TRAP staining showed minimal TRAP-positive signal and absence of multinucleated cell formation. Multinucleated cells are indicated by arrows. Scale bars = 100 μm (**A–E**).

The murine macrophage cell line RAW 264.7 was obtained as frozen cryovials from Cytion GmbH (Germany) and cultured in DMEM containing 10% FBS and 1% penicillin–streptomycin under standard conditions. Osteoclast differentiation was induced by supplementing the culture medium with macrophage colony-stimulating factor (M-CSF, 50 ng/mL; PeproTech, Rocky Hill, NJ, USA) and receptor activator of NF-κB ligand (RANKL, 50 ng/mL; PeproTech) for 5–7 days, following standard RAW 264.7 osteoclastogenesis methods [20, 21]. Differentiation was confirmed by TRAP staining, which showed multinucleated, TRAP-positive osteoclasts consistent with a mature osteoclastic phenotype (Fig. 2) [22]. Undifferentiated RAW 264.7 cells cultured without M-CSF and RANKL did not exhibit TRAP-positive staining.

#### Histological evaluation of chondrocyte spheroids

Histological staining was performed to confirm chondrogenic differentiation and ECM deposition in three-dimensional (3D) chondrocyte spheroids. After 21 days of differentiation, spheroids were harvested, washed twice with PBS (pH 7.4), and fixed in 4% paraformaldehyde for 20 min at room temperature. Fixed spheroids were embedded in 2% agarose, dehydrated through a graded ethanol series, cleared in xylene, and embedded in paraffin. Sections of 5 μm thickness were prepared using a rotary microtome (Leica Microsystems, Wetzlar, Germany) and mounted on poly-L-lysine-coated slides.

For Alcian Blue staining, sections were deparaffinized, rehydrated, and stained with 1% Alcian Blue 8GX (pH 2.5; Sigma-Aldrich, St. Louis, MO, USA) for 30 min to visualize sulfated glycosaminoglycans [23]. For Toluidine Blue staining, adjacent sections were stained with 0.1% Toluidine Blue (pH 4.1; Sigma-Aldrich) for 2–3 min to identify metachromatic proteoglycan-rich regions [23]. Hematoxylin and eosin (H&E) staining was performed according to standard procedures [24].

#### Tartrate-resistant acid phosphatase (TRAP) staining

TRAP staining was performed to confirm osteoclast differentiation from RAW 264.7 macrophages [25]. After 5–7 days of induction with M-CSF (50 ng/mL) and RANKL (50 ng/mL), cells were fixed with 4% paraformaldehyde for 15 min at room temperature and washed twice with PBS. TRAP activity was detected using an Acid Phosphatase, Leukocyte (TRAP) Kit (Sigma-Aldrich, St. Louis, MO, USA) according to the manufacturer’s protocol. Fixed cells were incubated with TRAP staining solution containing naphthol AS-BI phosphate and Fast Red Violet LB in the presence of 50 mM sodium tartrate for 1 h at 37 °C. TRAP-positive multinucleated cells exhibiting red–purple cytoplasmic staining were identified as mature osteoclasts and counted under a light microscope (Olympus BX43, Olympus, Tokyo, Japan). Differentiation efficiency was expressed as the percentage of TRAP-positive multinucleated cells relative to the total cell number per field.

### Co-culture of differentiated chondrocytes and osteoclasts

Differentiated chondrocytes and osteoclasts were directly co-cultured to allow cell–cell interaction and paracrine signaling and to investigate intercellular communication under shared culture conditions [26]. After lineage differentiation, both cell types were gently detached using TrypLE™ Express Enzyme (Thermo Fisher Scientific) for 2–3 min at 37 °C, with brief pipetting to facilitate release. Enzymatic activity was neutralized by adding an equal volume of complete medium containing 10% FBS. Cells were counted individually using a hemocytometer and resuspended in high-glucose DMEM supplemented with 10% FBS and 1% penicillin–streptomycin. Equal numbers of chondrocytes and osteoclast-like cells (1 × 10⁵ cells each per well) were combined to establish a 1:1 co-culture ratio.

Co-cultures were maintained at 37 °C in a humidified 5% CO₂ incubator. Culture medium was replaced every 24 h, and cells were maintained for up to 72 h for subsequent analyses. This direct co-culture configuration was selected to enable both contact-dependent and paracrine interactions between chondrocytes and osteoclasts under shared culture conditions. The model represents a simplified in vitro system to study osteochondral cell interactions rather than a full anatomical representation of the native joint microenvironment.

### Cell metabolic activity assay

Cellular metabolic activity of the chondrocyte–osteoclast co-culture was evaluated after 24 h and 48 h of incubation using the 3-(4,5-dimethylthiazol-2-yl)-2,5-diphenyltetrazolium bromide (MTT) assay (Sigma-Aldrich, St. Louis, MO, USA), a standard colorimetric method for assessing metabolic activity [27]. LPS was used as an experimental stimulus to model inflammatory stress in the co-culture system. Following LPS induction and HA treatment, culture medium was aspirated and cells were incubated with MTT solution (0.5 mg/mL in serum-free medium) for 4 h at 37 °C. Formazan crystals were dissolved in dimethyl sulfoxide (DMSO; 100 µL per well in 96-well plates or 1 mL per well in six-well plates), and absorbance was recorded at 570 nm using a microplate reader (BioTek Instruments, Winooski, VT, USA).

Cellular metabolic activity was expressed as a percentage relative to the untreated control group. All experiments were performed in triplicate and repeated independently at least three times. Cell area and morphology were analysed using ImageJ software with standard thresholding and segmentation methods.

### Morphological observation of co-cultured cells

Morphological changes in the chondrocyte–RAW 264.7 co-culture were examined using bright-field microscopy following LPS induction and HA treatment at different concentrations (50, 100, 200, and 500 µg/mL). After 24 h of LPS exposure, HA was added and cultures were incubated for a further 24 h. Morphology was observed under an inverted bright-field microscope (Olympus IX73, Olympus, Tokyo, Japan) at 10× and 20× magnification using identical illumination and exposure settings. Changes in cell shape, confluence, and overall structural integrity were recorded and qualitatively compared among untreated, LPS-treated, and HA-treated groups in three independent experiments.

### Western blot analysis

Protein expression of inflammatory and cartilage matrix-associated markers was analyzed by Western blot [28]. After treatment, cells were washed twice with cold PBS (pH 7.4) and lysed in radioimmunoprecipitation assay (RIPA) buffer (Thermo Fisher Scientific, Waltham, MA, USA) supplemented with protease and phosphatase inhibitor cocktail (Sigma-Aldrich, St. Louis, MO, USA). Lysates were incubated on ice for 30 min and centrifuged at 12,000 × g for 15 min at 4 °C, and the supernatant was collected. Protein concentrations were quantified using a bicinchoninic acid (BCA) assay kit (Thermo Fisher Scientific). Equal amounts of protein (20–40 µg) were mixed with Laemmli sample buffer, heated at 95 °C for 5 min, and separated by SDS–polyacrylamide gel electrophoresis using precast 6%, 8%, or 10% SDS–PAGE gels (Bio-Rad Laboratories) selected according to the molecular weight of the target proteins. Proteins were transferred onto nitrocellulose membranes (0.45 μm; Bio-Rad Laboratories, Hercules, CA, USA) at 100 V for 90 min. Membranes were horizontally sectioned according to molecular weight and sequentially stripped using mild stripping buffer for reprobing with different antibodies. After each stripping step, membranes were re-blocked and incubated with the appropriate primary and HRP-conjugated secondary antibodies. Densitometric analysis was performed using the loading control obtained during the initial probing prior to stripping. Molecular weights of detected proteins were verified using a standard protein ladder (10–250 kDa; Bio-Rad Precision Plus).

Membranes were blocked with 5% bovine serum albumin (BSA; Sigma-Aldrich) in Tris-buffered saline containing 0.1% Tween-20 (TBST) for 1 h at room temperature and incubated overnight at 4 °C with primary antibodies against NF-κB p65 (8242 S, Cell Signaling Technology, Danvers, MA, USA), phospho-NF-κB p65 (Ser536) (3033 S, Cell Signaling Technology), IL-1β (ab9722, Abcam, Cambridge, UK), TNF-α (ab6671, Abcam), MMP-13 (ab39012, Abcam), ADAMTS-5 (ab41037, Abcam), Collagen II (COL2A1; ab34712, Abcam), Aggrecan (ACAN; ab3778, Abcam), and GAPDH (ab8245, Abcam). After washing with TBST, membranes were incubated with appropriate horseradish peroxidase (HRP)-conjugated secondary antibodies (1:5000; Abcam) for 1 h at room temperature.

Bands were visualized using an enhanced chemiluminescence (ECL) detection system (Bio-Rad Laboratories) and imaged using the ChemiDoc™ MP Imaging System (Bio-Rad). Densitometric quantification of band intensity was performed using ImageJ software (NIH, Bethesda, MD, USA), and target protein expression was normalized to GAPDH.

### Quantitative real-time PCR (qPCR)

Total RNA was extracted from co-cultures using TRIzol™ reagent (Thermo Fisher Scientific, Waltham, MA, USA). RNA purity and concentration were verified by spectrophotometry at 260/280 nm using a NanoDrop™ 2000c spectrophotometer (Thermo Fisher Scientific). One microgram of total RNA was reverse-transcribed into cDNA using the High-Capacity cDNA Reverse Transcription Kit (Applied Biosystems, Foster City, CA, USA).

qPCR was performed using PowerUp™ SYBR™ Green Master Mix (Applied Biosystems) on a QuantStudio™ 5 Real-Time PCR System (Thermo Fisher Scientific) in a 20 µL reaction volume. Cycling conditions were: 95 °C for 10 min, followed by 40 cycles of 95 °C for 15 s and 60 °C for 60 s.

Specific primer pairs were designed for NF-κB p65, IL-1β, TNF-α, MMP-13, ADAMTS-5, Collagen II (COL2A1), Aggrecan (ACAN), and GAPDH (housekeeping control) (Table 1). Primer specificity and amplification efficiency (90–110%) were confirmed by melt-curve analysis [29]. Relative gene expression levels were calculated using the 2^–ΔΔCt method after normalization to GAPDH, with each experiment performed in triplicate from independent biological replicates (Table 1).

**Table 1:** Primer sequences used for quantitative real-time PCR (qPCR) analysis of inflammatory, catabolic, and extracellular matrix–associated genes in murine samples. Expression levels were normalized to GAPDH. Primer specificity was verified by BLAST analysis, and amplification efficiency (90–110%) was confirmed by melt-curve analysis. Data represent *n* = 3 independent biological replicates

Gene (Symbol)	Gene Name	Forward Primer (5′→3′)	Reverse Primer (5′→3′)	Accession No. (NCBI)	Amplicon Size (bp)
RELA	NF-κB p65	AGACCAAGGAGATGGACCTCA	CAGCCTTGTCCAGGAAGTCA	NM_009045.5	~ 120
IL1B	Interleukin-1β	GCAACTGTTCCTGAACTCAACT	ATCTTTTGGGGTCCGTCAACT	NM_008361.4	~ 130
TNF	Tumor necrosis factor-α	CCCTCACACTCAGATCATCTTCT	GCTACGACGTGGGCTACAG	NM_013693.3	~ 120
MMP13	Matrix metalloproteinase-13	CTGGCACACGCTTTTCCTCC	GATGCTGTCACCCACATCAGG	NM_008607.2	~ 140
ADAMTS5	A disintegrin and metalloproteinase with thrombospondin motifs 5	GTGCTCCACACATCTTCCAC	TGCTTGGCTGTCTTGGTGTT	NM_172860.3	~ 130
COL2A1	Collagen type II alpha 1	GACCCCATGCAGTACATGCG	ACGTCCTGGTGTCCTTGCAG	NM_031163.3	~ 150
ACAN	Aggrecan	AGTGGAGGTGATGGAAGCA	CGGTTGAGGTGTTGTCAGTT	NM_007424.3	~ 140
GAPDH	Glyceraldehyde-3-phosphate dehydrogenase	AGGTCGGTGTGAACGGATTTG	TGTAGACCATGTAGTTGAGGTCA	NM_008084.3	~ 120

### Enzyme-linked immunosorbent assay (ELISA) analysis

Concentrations of pro-inflammatory cytokines and matrix-degrading enzymes were quantified using mouse-specific ELISA kits (Abcam, Cambridge, UK) following established immunoassay procedures [30]. After treatment, culture supernatants were collected and centrifuged at 1,000 × g for 10 min at 4 °C to remove cell debris. Supernatants were aliquoted and stored at − 80 °C until analysis.

Levels of IL-1β (ab197742, Abcam), TNF-α (ab208348, Abcam), and MMP-13 (ab100605, Abcam) were measured according to the manufacturer’s protocols. In brief, 100 µL of standards or samples were added to antibody-coated wells and incubated for 2 h at room temperature. After washing, biotinylated detection antibodies were added for 1 h, followed by HRP-conjugated streptavidin for 30 min. Color development was achieved using TMB substrate, and reactions were stopped with 2 N sulfuric acid (H₂SO₄). Absorbance was measured at 450 nm using a microplate reader (BioTek Instruments). Concentrations were calculated from standard curves generated using recombinant standards. All assays were performed in triplicate, and results were expressed as pg/mL.

### Flow cytometric analysis of cell-cycle distribution

Cell-cycle distribution in the chondrocyte–osteoclast co-culture was analyzed by flow cytometry to evaluate changes in cellular proliferation following LPS and HA treatments. After incubation, cells were detached with TrypLE™ Express Enzyme (Thermo Fisher Scientific), washed twice with cold PBS, and fixed by gradual addition of ice-cold 70% ethanol while gently vortexing. Samples were stored at − 20 °C for at least 2 h, washed twice with PBS, and resuspended in propidium iodide (PI)/RNase A staining solution (50 µg/mL PI, 100 µg/mL RNase A in PBS). After 30 min at room temperature in the dark, DNA-content fluorescence was measured using a BD Accuri™ C6 Plus flow cytometer (BD Biosciences, San Jose, CA, USA). At least 20,000 singlet events per sample were acquired. Data were analyzed with FlowJo v10 software (Tree Star, Ashland, OR, USA) to quantify the proportions of cells in G₀/G₁, S, and G₂/M phases [31].

###  DNA-fragmentation assay

DNA fragmentation was examined to assess changes in genomic integrity under inflammatory conditions. After treatment, genomic DNA was isolated using a GenElute™ Mammalian Genomic DNA Miniprep Kit (Sigma-Aldrich) according to the manufacturer’s instructions. DNA (2 µg per sample) was mixed with 6× loading dye and resolved on a 1.8% agarose gel containing ethidium bromide (0.5 µg/mL) at 90 V for 1.5 h in 1× TAE buffer. Gels were visualized under a UV transilluminator (Gel Doc™ XR+, Bio-Rad Laboratories), and images were recorded. Continuous high-molecular-weight bands indicated intact genomic DNA, whereas smeared or ladder-like patterns reflected fragmentation [32]. Band-intensity profiles were quantified using ImageJ (NIH, USA), and data were expressed as relative fragmentation units (arbitrary units) normalized to the control group.

### Statistics

All experiments were conducted using three independent biological replicates per condition. MTT and ELISA assays were performed in technical triplicates and averaged within each biological replicate. Data are presented as mean ± standard deviation (SD). Statistical comparisons were performed using one-way analysis of variance (ANOVA) followed by Tukey’s multiple-comparison post hoc test. Differences were considered statistically significant at *p* < 0.05 [33].

## Results

### Validation of chondrogenic differentiation

Differentiated chondrocytes exhibited positive Alcian Blue and Toluidine Blue staining, indicating proteoglycan-rich matrix formation (Fig. 1). In addition, the expression of COL2A1 and ACAN at both mRNA and protein levels supported acquisition of a cartilage-like phenotype following differentiation.

### Effects of HA on cellular metabolic activity and morphology

**Fig. 3:**
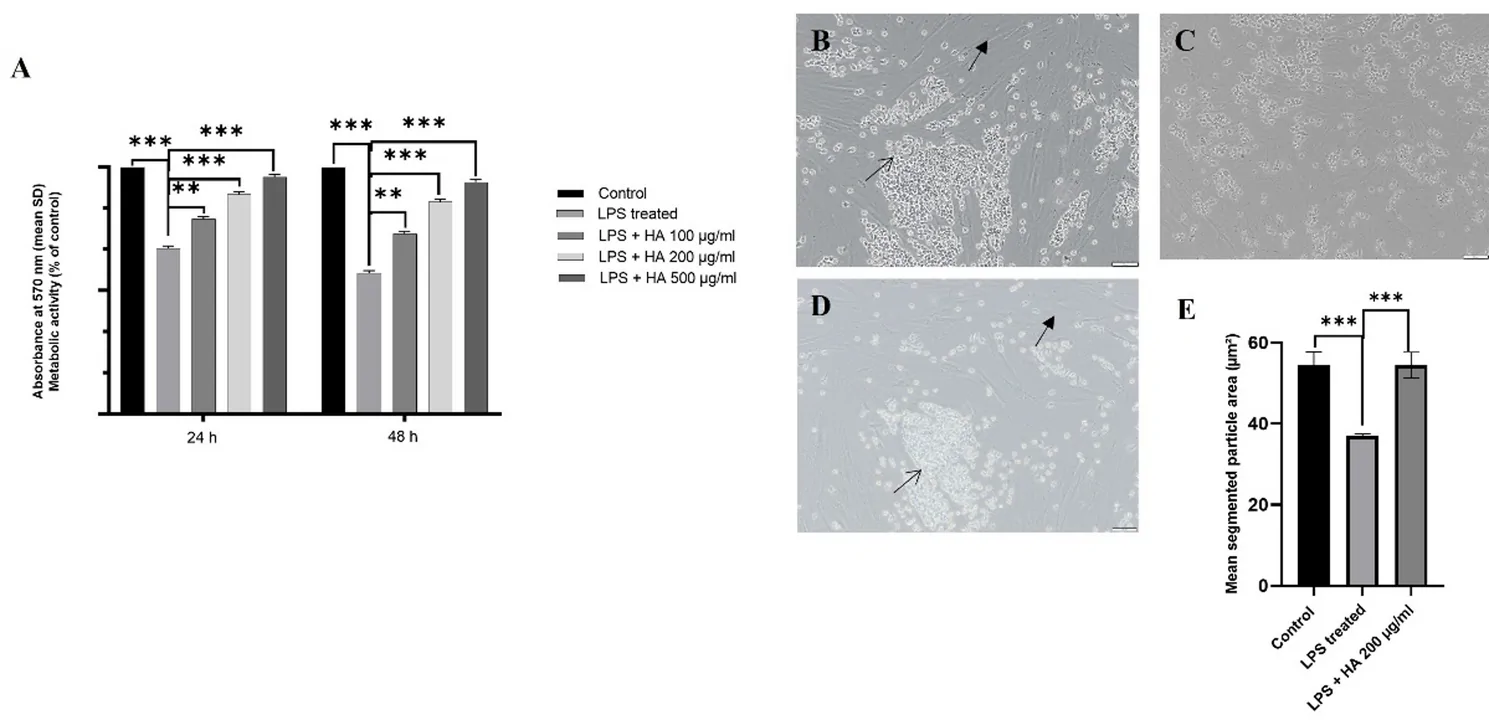
Cellular metabolic activity and morphological observation of chondrocyte–osteoclast co-cultures. (**A**) MTT assay showing metabolic activity (% of control) after 24 h and 48 h of LPS stimulation and HA treatment (50–500 µg/mL). Data are presented as mean ± SD (*n* = 3) and analyzed by one-way ANOVA with Tukey’s post-hoc test. (B–D) Bright-field micrographs of co-cultures under the indicated conditions. (**B**) Co-cultures showed typical cell morphology. (**C**) LPS stimulation was associated with reduced cell spreading and increased cell detachment. (**D**) HA treatment (200 µg/mL) partially restored cell spreading and increased cell density relative to the LPS-treated group. Scale bars = 100 μm (**B–D**). (**E**) Quantification of mean segmented particle area (µm²) derived from Fiji/ImageJ-based analysis. Identical thresholding and particle-analysis parameters were applied across all groups. LPS treatment significantly reduced segmented particle area compared to control, indicating loss of cellular spreading and structural integrity, whereas HA treatment significantly restored these parameters compared to the LPS-treated group. Data are presented as mean ± SD (*n* = 3 independent experiments). Statistical significance: ***p < 0.001.

Cellular metabolic activity in the chondrocyte–osteoclast co-culture was evaluated following LPS induction and HA treatment for 24 h and 48 h (Fig. 3). LPS significantly reduced metabolic activity relative to the untreated control (*p* < 0.05), consistent with the induction of inflammatory stress. HA treatment increased metabolic activity in a concentration-dependent manner, with maximal values observed at 200–500 µg/mL (*p* < 0.001). Metabolic activity at 500 µg/mL did not show further improvement beyond 200 µg/mL, suggesting a concentration plateau under the present conditions.

Metabolic activity declined slightly at 48 h, which may reflect prolonged culture-associated metabolic decline rather than HA-associated cytotoxicity.

Bright-field microscopy (Fig. 3) showed organized chondrocyte morphology and multinucleated osteoclasts in control cultures. LPS exposure resulted in morphological alterations, including reduced spreading and partial detachment. HA treatment attenuated these changes, and cells in HA-treated groups appeared more similar to control cultures than to LPS-treated cultures, suggesting improved cellular architecture under inflammatory conditions. These observations represent overall responses of the mixed cell population in the co-culture system.

### Western blot analysis of inflammatory and matrix-regulatory proteins

**Fig. 4:**
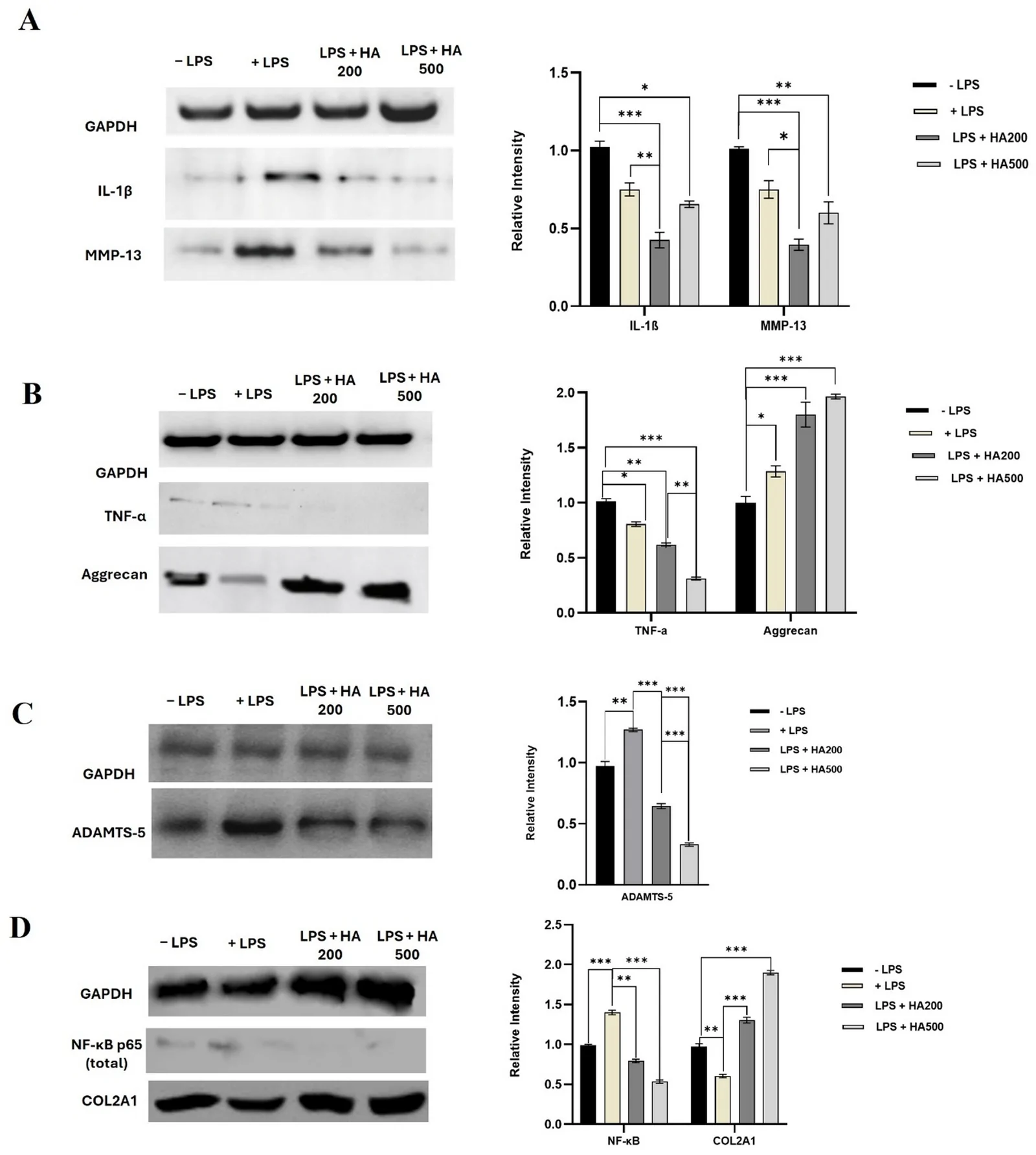
Hyaluronic acid modulates inflammatory and matrix-regulatory pathways in LPS-stimulated chondrocyte–osteoclast co-culture. (**A**) Representative Western blots and densitometric quantification of IL-1β and MMP-13 expression following lipopolysaccharide (LPS, 1 µg/mL) stimulation and treatment with high-molecular-weight hyaluronic acid (HA; 200 and 500 µg/mL). LPS increased IL-1β and MMP-13 levels relative to the non-stimulated control, consistent with inflammatory and catabolic responses. HA co-treatment was associated with reduced expression of both markers. (**B**) Immunoblot analysis of TNF-α and Aggrecan (ACAN). LPS increased TNF-α expression and reduced Aggrecan abundance. HA treatment was associated with reduced TNF-α levels and partial restoration of Aggrecan expression. (**C**) Western blot and densitometric analysis of ADAMTS-5 expression. LPS increased ADAMTS-5 levels, whereas HA treatment was associated with reduced expression. (**D**) Representative immunoblots for total NF-κB p65 and COL2A1. LPS increased NF-κB p65 and reduced COL2A1 expression. HA treatment was associated with reduced NF-κB p65 levels and restoration of COL2A1 expression. All blots were normalized to GAPDH as an internal loading control. Bar graphs represent mean ± SD (*n* = 3). Statistical comparisons were performed using one-way ANOVA with Tukey’s post-hoc test (*p < 0.05, **p < 0.01, ***p < 0.001, ****p < 0.0001). Approximate molecular weights: IL-1β (~ 31 kDa), TNF-α (~ 17 kDa), MMP-13 (~ 54 kDa), ADAMTS-5 (~ 75 kDa), NF-κB p65 (~ 65 kDa), COL2A1 (~ 130–140 kDa), GAPDH (~ 36 kDa)

Western blotting was performed to evaluate the effects of HA on inflammatory markers and extracellular-matrix regulators (Figs. 4). LPS exposure increased protein levels of NF-κB p65, phospho-NF-κB p65, IL-1β, and TNF-α, indicating enhanced inflammatory responses under the present conditions. Concomitantly, the matrix-degrading enzymes MMP-13 and ADAMTS-5 were elevated, while COL2A1 and ACAN were reduced, reflecting a shift toward catabolic conditions.

HA treatment decreased LPS-associated increases in NF-κB p65, IL-1β, and TNF-α in a concentration-dependent manner and reduced MMP-13 and ADAMTS-5 levels. COL2A1 and ACAN expression was higher in HA-treated groups relative to LPS alone. At 500 µg/mL, band intensities were broadly comparable to those at 200 µg/mL, suggesting a concentration plateau.

Collectively, these findings indicate that HA treatment was associated with a relative reduction in inflammatory and catabolic protein expression alongside preservation of ECM-associated markers.

### Quantitative real-time PCR (qPCR) analysis

**Fig. 5:**
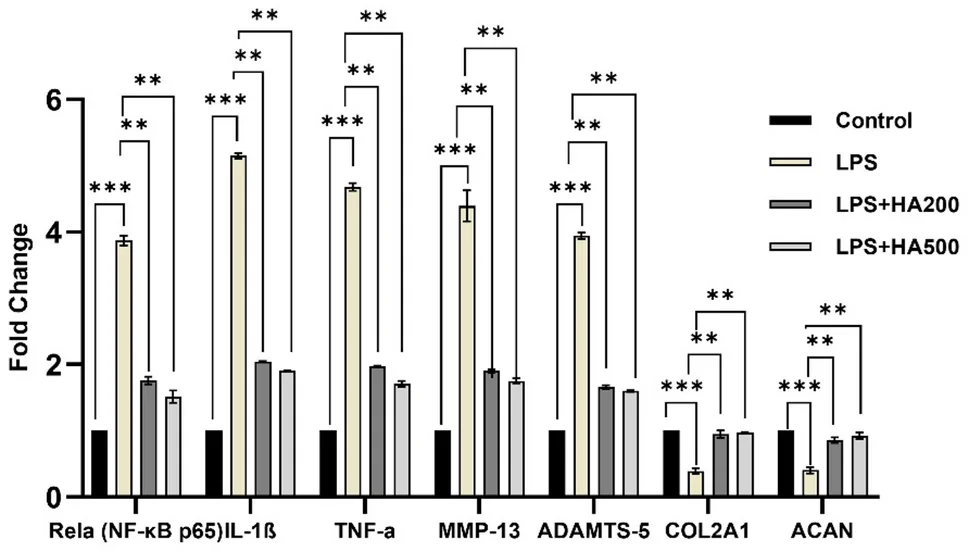
Quantitative real-time PCR (qRT-PCR) analysis of inflammatory, catabolic, and anabolic gene expression in LPS-stimulated chondrocyte–osteoclast co-culture following hyaluronic acid (HA) treatment. Relative mRNA expression levels of RELA (NF-κB p65), IL1B, TNF, MMP13, ADAMTS5, COL2A1, and ACAN were quantified in control, LPS-treated, and HA-treated groups (LPS + HA 200 µg/mL and LPS + HA 500 µg/mL). LPS stimulation was associated with increased expression of inflammatory (RELA, IL1B, TNF) and catabolic (MMP13, ADAMTS5) genes, along with reduced expression of extracellular matrix–associated genes (COL2A1, ACAN). HA treatment was associated with reduced expression of inflammatory and catabolic transcripts and partial recovery of COL2A1 and ACAN expression toward control levels. Data are expressed as fold change (2⁻ΔΔCt) relative to the control group after normalization to GAPDH. Values represent mean ± SD (*n* = 3 biological replicates), with each sample analyzed in technical triplicates. Statistical analysis was performed using one-way ANOVA followed by Tukey’s post-hoc test (*p < 0.05, **p < 0.01, ***p < 0.001; ns = not significant)

mRNA expression analysis demonstrated that LPS significantly increased Rela, Il1b, and Tnf, along with the catabolic enzymes Mmp13 and Adamts5 (*p* < 0.01), while reducing Col2a1 and Acan expression (Fig. 5). HA treatment at 200 and 500 µg/mL was associated with lower expression of inflammatory and catabolic transcripts and higher levels of anabolic genes compared with LPS alone.

These reciprocal expression patterns were consistent with the Western blot results and suggest a relative shift toward ECM-associated gene expression under the present experimental conditions. Expression values were comparable between 200 and 500 µg/mL, in line with Western blot observations and consistent with a concentration plateau in this experimental setting.

### Cell-cycle distribution and DNA-integrity analysis

**Fig. 6:**
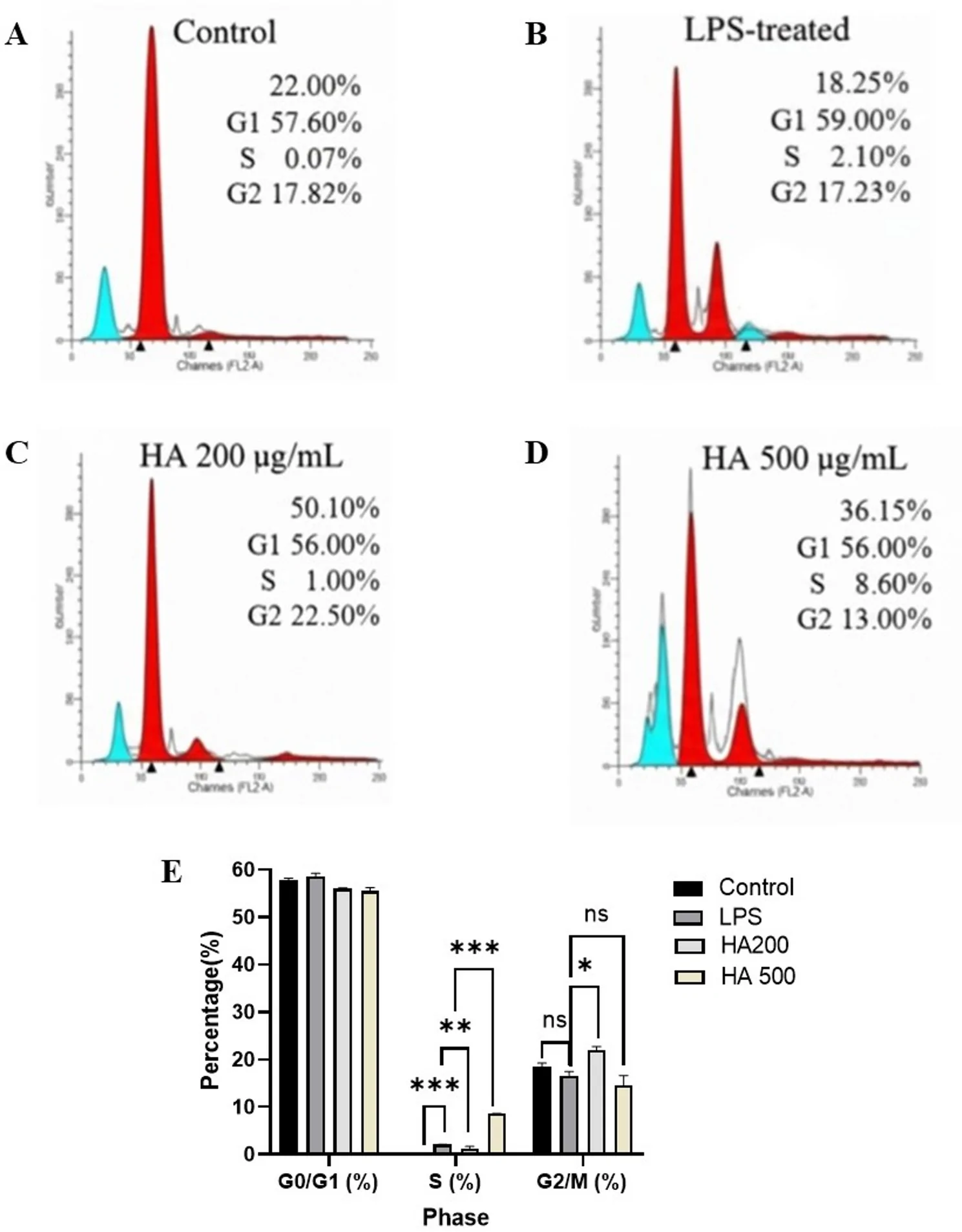
Flow-cytometric analysis of cell-cycle distribution in chondrocyte–osteoclast co-cultures following LPS stimulation and hyaluronic acid (HA) treatment. (**A–D**) Representative DNA-content histograms showing the distribution of cells across the G0/G1, S, and G2/M phases after 24 h under the indicated conditions: (**A**) untreated control, (**B**) LPS-treated (1 µg/mL), (**C**) LPS + HA 200 µg/mL, and (**D**) LPS + HA 500 µg/mL. Cells were fixed in 70% ethanol, stained with propidium iodide (PI) in the presence of RNase A, and analyzed using a BD Accuri™ C6 Plus flow cytometer. DNA-histogram deconvolution and phase quantification were performed using FlowJo v10 software. LPS exposure was associated with accumulation of cells in the G0/G1 phase and alterations in S-phase distribution. HA treatment was associated with changes in cell-cycle distribution, including increased S-phase and G2/M fractions relative to LPS-treated cells. (**E**) Quantitative analysis of cell-cycle distribution expressed as mean ± SD from three independent biological replicates (*n* = 3). Statistical analysis was performed using one-way ANOVA followed by Tukey’s post-hoc test (*p < 0.05, **p < 0.01, ***p < 0.001; ns = not significant)

Flow-cytometric analysis showed that LPS stimulation increased the proportion of cells in G₀/G₁ and reduced S-phase populations, suggesting altered cell-cycle distribution under inflammatory conditions (Fig. 6). HA-treated groups demonstrated a partial shift toward distributions more similar to control conditions, with increased S-phase fractions at 200 and 500 µg/mL. These findings represent population-level responses within the co-culture system and do not distinguish between individual cell types.

**Fig. 7:**
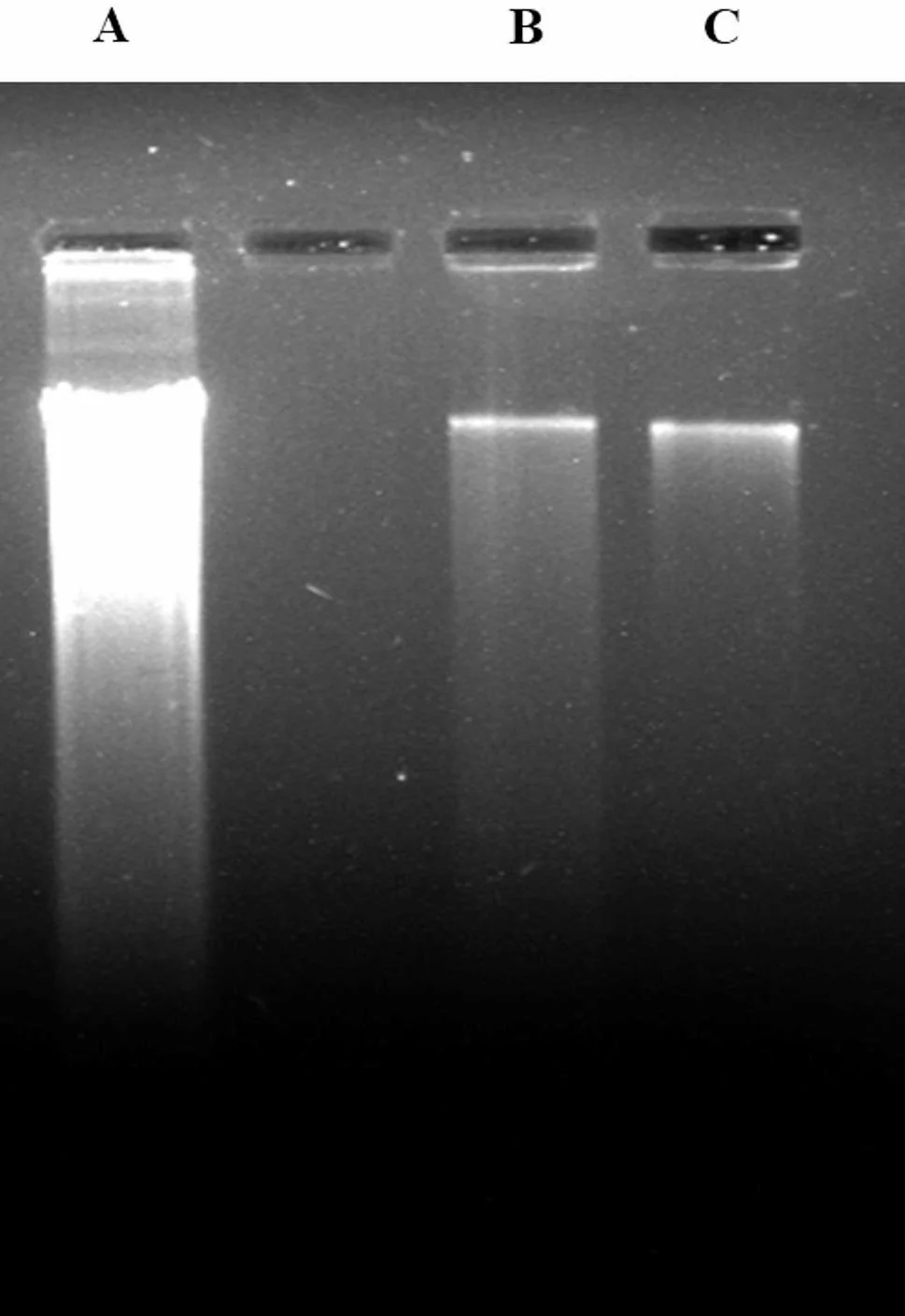
Hyaluronic acid attenuates LPS-induced DNA fragmentation in chondrocyte–osteoclast co-culture. Representative agarose gel electrophoresis image (1.8% agarose, ethidium bromide stained) showing genomic DNA fragmentation in LPS-stimulated cells and following HA treatment. (**A**) LPS-treated group showing intense DNA smearing and degraded low-molecular-weight fragments, consistent with increased DNA fragmentation under inflammatory stress. (**B**) LPS + HA 200 µg/mL group showing partial reduction in DNA smearing, with a more compact and higher-molecular-weight band pattern, suggesting moderate protection against LPS-induced genotoxicity. (**C**) LPS + HA 500 µg/mL group showing a prominent intact DNA band with minimal smearing, reflecting substantial restoration of genomic integrity and reduced DNA smearing relative to the LPS-treated group following HA treatment

DNA-fragmentation analysis (Fig. 7) showed increased DNA smearing in LPS-treated samples, whereas HA-treated groups exhibited comparatively more intact genomic DNA bands. These observations reflect qualitative differences in DNA integrity under the present experimental conditions.

### ELISA quantification of secreted cytokines and matrix-degrading enzymes

**Fig. 8:**
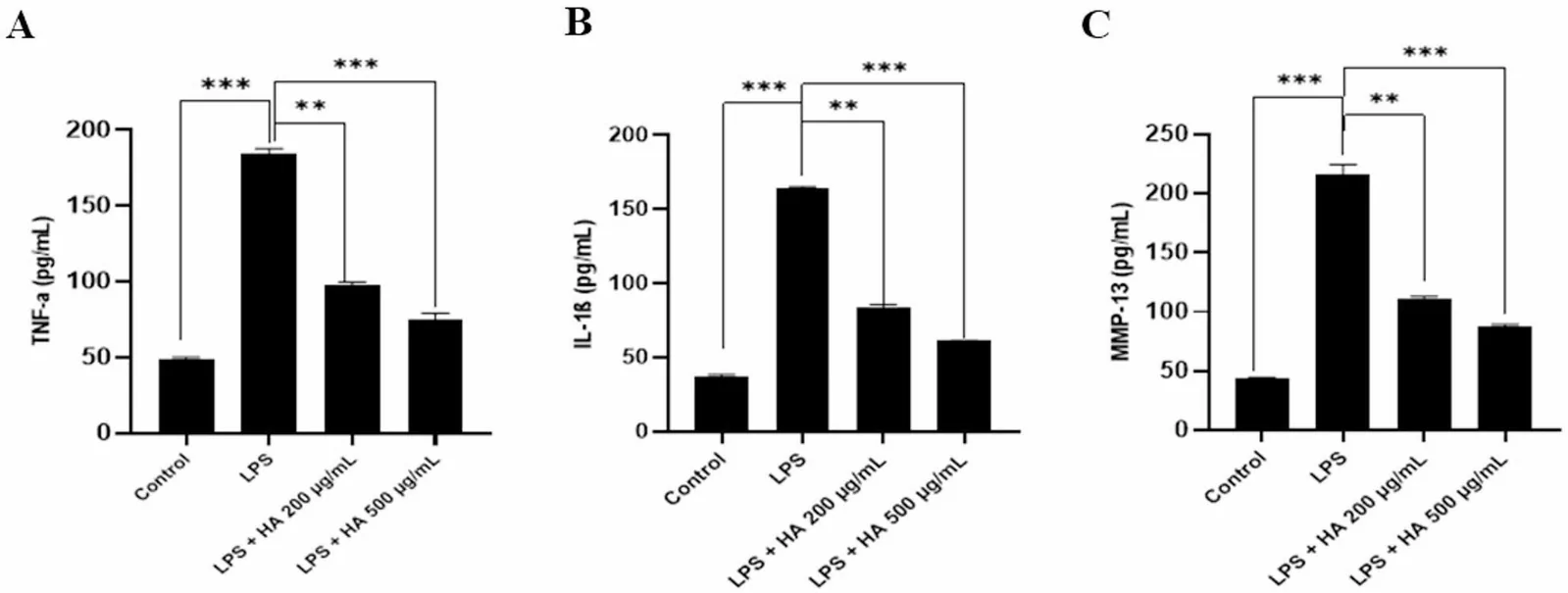
Quantitative ELISA analysis of inflammatory cytokine and matrix-degrading enzyme secretion in LPS-stimulated chondrocyte–osteoclast co-culture following hyaluronic acid (HA) treatment. (**A**) TNF-α, (**B**) IL-1β, and (**C**) MMP-13 concentrations in culture supernatants were quantified by ELISA under control, LPS, and HA treatment conditions (LPS + HA 200 µg/mL; LPS + HA 500 µg/mL). LPS exposure markedly increased cytokine and protease secretion compared with the control group, consistent with an inflammatory and catabolic response. HA treatment reduced secretion of these mediators, with lower levels observed at 500 µg/mL than at 200 µg/mL, approaching but not fully returning to control levels. Data represent mean ± SD of three independent biological replicates (*n* = 3). Statistical analysis was performed using one-way ANOVA followed by Tukey’s post-hoc test (**p < 0.01, ***p < 0.001)

ELISA analysis showed increased secretion of IL-1β, TNF-α, and MMP-13 in LPS-exposed co-cultures relative to controls (Fig. 8). HA treatment reduced secretion of these mediators in a concentration-dependent manner, with 500 µg/mL showing values approaching those of untreated controls. These findings were consistent with the Western blot and qPCR data and indicate that HA treatment was associated with lower inflammatory and catabolic mediator levels under the present in-vitro conditions.

## Discussion

OA is a chronic degenerative joint disorder characterised by progressive cartilage erosion, synovial inflammation, and subchondral bone remodeling [34, 35]. The pathogenesis is influenced by pro-inflammatory cytokines, oxidative stress, and enzymatic matrix degradation, creating conditions that can support an inflammatory microenvironment [36]. Cell–matrix interactions and mechanotransduction pathways, including integrin-mediated signaling, also play critical roles in chondrocyte function and cartilage homeostasis [37]. Crosstalk between chondrocytes and osteoclasts is considered an important contributor to this process, as osteoclast-derived factors may influence chondrocyte catabolic responses. In the present study, both cell types were included in a co-culture system; however, the specific contribution of osteoclast-like cells to the observed responses cannot be independently distinguished under the current experimental conditions. The co-culture system used in this study enables evaluation of combined cellular responses under inflammatory conditions, which cannot be fully captured in monoculture systems [38, 39].

### Hyaluronic acid and the inflammatory microenvironment

The HA used in the present study represents a stabilized, crosslinked formulation rather than native linear hyaluronic acid. Hyaluronic acid is an endogenous component of synovial fluid and has been widely recognized as a key regulator of inflammatory processes and immune responses, influencing cellular signaling, oxidative balance, and inflammatory mediators [40–42]. Previous studies describe HMW HA interactions with CD44 and RHAMM, which have been reported to be associated with intracellular signaling pathways including PI3K/Akt, ERK1/2, and p38 MAPK, as well as TLR4/NF-κB signaling; these mechanisms were not directly assessed in the present study [43, 44]. However, the precise contribution of these pathways in complex cellular systems remains an active area of investigation.

In the present study, LPS was used as a controlled stimulus to induce inflammatory signaling under in-vitro conditions. This represents an acute inflammatory model and does not fully reflect the chronic low-grade inflammation observed in osteoarthritis. LPS exposure was associated with reduced cellular metabolic activity and altered morphology in the co-culture model, consistent with reports that LPS–TLR4 engagement can trigger mitochondrial dysfunction and inflammatory stress [45, 46]. HA supplementation at 100–200 µg/mL showed partial improvement in viability and morphology, while 500 µg/mL did not demonstrate a further increase beyond this range, suggesting an upper concentration effect under the present experimental conditions [47]. These observations suggest that the HA concentrations used in this model were cytocompatible under the present experimental conditions.

### Inflammatory and ECM-regulatory pathways

In Western blot and qPCR analyses, LPS exposure was associated with increased expression of NF-κB p65 and downstream inflammatory markers, while HA treatment was associated with lower expression of these markers. This pattern is consistent with previous studies reporting that HMW HA is associated with modulation of inflammatory transcription and NF-κB-related signaling pathways [48–51]. However, these mechanisms were not directly assessed in the present study and therefore are interpreted as associations rather than direct pathway modulation. At the same time, anabolic markers COL2A1 and ACAN demonstrated higher expression in HA-treated conditions than in LPS alone, indicating a relative shift toward extracellular-matrix associated gene expression. Such reciprocal expression patterns agree with previous reports describing potential roles of HA in inflammatory modulation and ECM-related gene regulation [52, 53].

### Secreted mediators

ELISA data revealed that LPS increased secretion of IL-1β, TNF-α, and MMP-13, while HA treatment was associated with lower levels of these mediators. These findings parallel intracellular expression data and are in line with earlier reports that HMW HA may influence both intracellular transcription and extracellular mediator dynamics [54].

Cytokine output is closely linked to inflammatory transcriptional activity and intracellular trafficking pathways in macrophage-derived cells [54, 55].

### Genome and cell-cycle observations

LPS-treated groups demonstrated G0/G1 accumulation together with increased DNA fragmentation, whereas HA-treated groups showed comparatively more balanced cell-cycle distributions and less DNA smearing under the present conditions. These findings indicate relative differences in genomic integrity and cell-cycle distribution; however, they should be interpreted cautiously because the analyses were performed in a mixed co-culture population and do not allow attribution to individual cell types. In addition, the DNA-fragmentation assay is qualitative and does not provide a direct quantitative measure of apoptosis [56–58].

### Translational relevance

Collectively, these observations provide molecular-level information regarding HA-associated changes in inflammatory and ECM-related markers under the present co-culture conditions. The consistent patterns observed across Western blot, qPCR, ELISA, and cell-cycle analyses support a model in which HA treatment coincides with reduced inflammatory and catabolic signals, alongside higher levels of ECM-associated markers. While these findings are based on an in-vitro model, they align with prior work suggesting potential relevance of HMW HA to OA-related inflammatory mechanisms. However, further mechanistic studies, 3D organoid models, and in-vivo systems will be needed to clarify the extent and durability of these effects and to determine their translational significance. Nevertheless, crosslinked HA formulations are known to retain biological activity, including modulation of inflammatory and extracellular matrix-associated responses. These findings are consistent with clinical evidence indicating that intra-articular hyaluronic acid exerts therapeutic effects through both mechanical and molecular mechanisms [59]. Systematic reviews have further demonstrated its efficacy in improving osteoarthritis symptoms [60], while recent studies highlight its role in modulating inflammatory pathways and joint function [61].

## Limitations of the study

This study has several limitations. The experiments were performed in an in-vitro chondrocyte–osteoclast co-culture model, which cannot fully represent the complexity of the osteoarthritic joint environment in vivo. Additional joint components such as synoviocytes, immune cells, and biomechanical loading were not included. The use of RAW 264.7-derived osteoclast-like cells and MSC-derived chondrocytes represents a simplified model system and may not fully replicate primary human cell behavior. In addition, the direct co-culture design does not fully recapitulate the spatial organization of chondrocytes and osteoclasts in vivo and should be interpreted as a simplified model of osteochondral cell interactions. Osteoclast differentiation was generated from the RAW 264.7 murine macrophage cell line, which may not completely reflect the behavior of primary human osteoclast precursors. Inflammation was induced using lipopolysaccharide (LPS) as an experimental stimulus, which models acute inflammatory activation rather than the chronic low-grade inflammation typical of osteoarthritis. Although multiple molecular approaches, including Western blotting, qPCR, ELISA, and flow cytometry, were used to evaluate inflammatory and extracellular matrix markers, the study mainly identified associations between hyaluronic acid treatment and molecular changes rather than confirming direct signaling mechanisms. In addition, the experiments were conducted over short treatment periods, which limits evaluation of long-term cellular responses and matrix remodeling. Therefore, further studies using advanced three-dimensional models and in vivo systems are required to validate the translational relevance of these findings. As the HA used in this study was a crosslinked formulation, structural modification may influence its physicochemical properties and cellular interactions compared to native hyaluronic acid, and therefore the findings should be interpreted in the context of the specific formulation used. Hyaluronidase activity and potential degradation of HA were not assessed in this study and therefore cannot be excluded, which may influence HA bioactivity [62]. In addition, lineage-specific transcription factors such as SOX9, which regulates chondrogenic differentiation, and hypertrophic markers including RUNX2 and type X collagen (COL10A1) were not assessed in the present study. Therefore, the distinction between stable chondrogenic differentiation and hypertrophic maturation cannot be fully established, as these pathways are known to differentially regulate cartilage development and hypertrophic progression [63, 64].

The use of a single cell source does not account for donor-to-donor variability, which may influence cellular responses.

Despite these limitations, the present study provides important insight into the modulatory effects of high-molecular-weight hyaluronic acid on inflammatory signaling and extracellular matrix regulation within a chondrocyte–osteoclast co-culture system.

## Conclusion

This study investigated the effects of high-molecular-weight hyaluronic acid (HMW-HA) in an inflammatory chondrocyte–osteoclast co-culture system designed to model key features of osteoarthritic degeneration. Under the present in-vitro conditions, HA treatment was associated with reduced NF-κB-related inflammatory signaling and lower expression of pro-inflammatory cytokines and matrix-degrading enzymes, including IL-1β, TNF-α, MMP-13, and ADAMTS-5. In parallel, HA-treated cultures demonstrated comparatively higher expression of extracellular matrix–associated markers, such as COL2A1 and ACAN.

These coordinated molecular changes suggest that HA treatment coincided with a relative reduction in inflammatory and catabolic signaling alongside relative preservation of ECM-related markers in this experimental system. The consistent patterns observed across Western blot, qPCR, ELISA, and cell-cycle analyses support a model in which HA may contribute to maintaining cellular homeostasis under inflammatory conditions.

Overall, these findings provide molecular evidence describing HA-associated modulation of inflammatory and extracellular-matrix-related pathways in a chondrocyte–osteoclast co-culture model of osteoarthritis. Although the study was conducted under controlled in-vitro conditions, the results contribute to the growing understanding of HA-related biological activity in inflammatory joint environments. Further mechanistic studies using advanced three-dimensional models and in-vivo systems will be required to clarify the underlying pathways and determine the translational significance of these observations.

## Supplementary Information

Below is the link to the electronic supplementary material.

Supplementary Material 1: Supplementary Western blot data (PDF). Contains original membrane regions corresponding to immunoblot analyses presented in Fig. 4.

Supplementary Material 2: Supplementary data for Fig. 3 (PDF). Contains additional quantitative analysis and/or supporting images related to cellular metabolic activity and morphological observations in the chondrocyte–osteoclast co-culture.

## Data Availability

The datasets generated and analyzed during the current study are available from the corresponding author upon reasonable request.

## Declarations

### Clinical trial number

Clinical trial number: Not applicable.

## References

[1] Martel-Pelletier J, Barr AJ, Cicuttini FM, Conaghan PG, Cooper C, Goldring MB, Goldring SR, Pelletier JP. Osteoarthritis. Nat Reviews Disease Primers. 2021;7(1):5. 10.1038/nrdp.2016.72 27734845

[2] Hunter DJ, Bierma-Zeinstra S. Osteoarthr Lancet. 2019;393(10182):1745–59. 10.1016/S0140-6736(19)30417-9 31034380

[3] Safiri S, Kolahi AA, Smith E, Hill C, Bettampadi D, Mansournia MA, Hoy D, Ashrafi-Asgarabad A. Global burden of osteoarthritis 1990–2019. Ann Rheum Dis. 2020;79(6):819–28. 10.1136/annrheumdis-2019-216515 32398285

[4] Cui A, Li H, Wang D, Zhong J, Chen Y, Lu H. Global, regional prevalence and incidence of osteoarthritis. Osteoarthr Cartil. 2020;28(2):154–63.

[5] Charlier E, Relic B. Chondrocyte dysfunction in osteoarthritis. Nat Rev Rheumatol. 2021;17(1):51–71.

[6] Li Y, Xiao J, Wang Y, Luo S, Zhang H, Zhang X. IL-1β and TNF-α activate NF-κB–mediated cartilage degradation pathways. J Cell Mol Med. 2021;25(1):488–500.

[7] Zhou Y, Wang T, Hamilton JL, Chen D. Roles of MMP-13 and ADAMTS-5 in cartilage matrix degradation. Bone Res. 2022;10(1):12.

[8] Zhen G, Wen C, Jia X, Li Y, Crane JL, Mears SC, Cao X. (2021) Chondrocyte–osteoclast crosstalk in osteoarthritis. Nat Commun. 12(1):547.

[9] Fu K, Robbins SR, McDougall J, Barr AJ. Osteoclast-driven subchondral bone remodelling in osteoarthritis. Bone. 2022;157:116327.

[10] Kloppenburg M, Berenbaum F. Osteoarthritis: current treatment and future perspectives. Lancet. 2020;395(10242):171–81.

[11] Larrañaga-Vaquero F, García-Aznar JM, Ballester-Beltrán J, Baaijens F, Oyen M. Hyaluronic acid in synovial joint biomechanics. Biomaterials. 2024;301:122273.

[12] Altman RD, Manjoo A. Mechanical and viscoelastic properties of hyaluronic acid in the joint. Osteoarthr Cartil. 2021;29(1):1–12.

[13] Basu S, Nguyen QT, Montel L, Basu S, Choi AJ, Fong Y. CD44- and TLR4-mediated hyaluronic acid signalling regulates inflammatory responses. J Biol Chem. 2020;295(2):484–97.

[14] Miyamoto K, Yamaguchi H, Tamura M, Sato S. Molecular- weight–dependent biological effects of hyaluronic acid in osteoarthritis. Front Bioeng Biotechnol. 2021;9:p703823.

[15] Zhang L, Zhang Y, Li Y, Zhang X. Low-molecular-weight hyaluronic acid promotes inflammation and matrix degradation. Carbohydr Polym. 2023;304:120607.

[16] García-García M, Sánchez-Montero JM, Casas J. High-molecular-weight hyaluronic acid suppresses NF-κB activation and pro-inflammatory cytokines in chondrocytes. Int J Mol Sci. 2022;23(6):3311.

[17] Dai X, Gao L, Li Y, Wang S, Xu Y. Hyaluronic acid enhances type II collagen and aggrecan synthesis in human chondrocytes. Biomaterials Sci. 2023;11(7):2101–14.

[18] Johnstone B, et al. Chondrocyte differentiation from mesenchymal stem cells. Stem Cells. 2020;38(4):485–97.

[19] Kim M, Im GI. Spheroid culture enhances chondrogenic potential of mesenchymal stem cells. Biomaterials. 2021;269:120527.

[20] Park JH, et al. Osteoclastogenesis from RAW 264.7 macrophages using RANKL stimulation. J Cell Physiol. 2021;236(2):1558–69.

[21] Zhang Y, Huang H. M-CSF/RANKL-induced osteoclast differentiation model. Front Cell Dev Biol. 2022;10:841012.

[22] Takahashi N, et al. Tartrate-resistant acid phosphatase as a marker for osteoclast maturation. Bone. 2020:136;115330.

[23] Caron MMJ, et al. Histological markers of chondrogenesis: Alcian Blue and Toluidine Blue staining for proteoglycans. Osteoarthr Cartil. 2020;28(3):365–77.

[24] Fischer AH, Jacobson KA, Rose J, Zeller R. Hematoxylin and eosin staining of tissue and cell sections. Cold Spring Harbor Protoc. 2008;3(5):pppdbprot4986. 10.1101/pdb.prot4986 21356829

[25] Takahashi N, Udagawa N, Suda T. Tartrate-resistant acid phosphatase as a marker for osteoclasts. Bone. 2020:136:115330.

[26] Chen X, Su Y, Xu Q, Wang H, Li Z. Chondrocyte–osteoclast co-culture models to study intercellular interactions in osteoarthritis. J Orthop Res. 2021;39(5):1010–20.

[27] Mosmann T. Rapid colorimetric assay for cellular growth and survival: application to proliferation and cytotoxicity assays. J Immunol Methods. 1983;65(1–2):55–63. 10.1016/0022-1759(83)90303-4 6606682

[28] Mahmood T, Yang PC. Western blot: technique, theory and troubleshooting. J Visual Exp. 2012;63:e3578.

[29] Livak KJ, Schmittgen TD. Analysis of relative gene expression data using real-time quantitative PCR and the 2^–∆∆Ct method. Methods. 2001;25(4):402–8. 10.1006/meth.2001.1262 11846609

[30] Crowther JR. The ELISA Guidebook. New York: Humana; 2020.

[31] Darzynkiewicz et al. Methods to analyze cell cycle by flow cytometry (Method in Molecular Biology; a gold-standard reference). 2018.

[32] Wyllie AH. Apoptosis: DNA fragmentation as a biochemical hallmark. Nature. 1980;284(5756):555–6. 10.1038/284555a0 6245367

[33] Motulsky H. Intuitive Biostatistics: A Nonmathematical Guide to Statistical Thinking. 4th ed. New York: Oxford University Press; 2021.

[34] Martel-Pelletier J, Barr AJ, Cicuttini FM, et al. Osteoarthritis. Nat Reviews Disease Primers. 2016;2:16072. 10.1038/nrdp.2016.72 27734845

[35] Glyn-Jones S, Palmer AJR, Agricola R, et al. Osteoarthritis. Lancet. 2015;386(9991):376–87. 10.1016/S0140-6736(14)60802-3 25748615

[36] Kapoor M, Martel-Pelletier J, Lajeunesse D, Pelletier J-P, Fahmi H. Role of pro-inflammatory cytokines in the pathophysiology of osteoarthritis. Nat Rev Rheumatol. 2011;7(1):33–42. 10.1038/nrrheum.2010.196 21119608

[37] Millward-Sadler SJ, Salter DM. Integrin-dependent signal cascades in chondrocyte mechanotransduction. Ann Biomed Eng. 2004;32(3):435–46. 10.1023/b:abme.0000017538.72511.48 15095818

[38] Goldring MB, Goldring SR. Articular cartilage and subchondral bone in the pathogenesis of osteoarthritis. Ann N Y Acad Sci. 2010;1192(1):230–7. 10.1111/j.1749-6632.2009.05240.x 20392241

[39] Schett G, Teitelbaum SL. Osteoclasts and arthritis. J Bone Miner Res. 2009;24(7):1142–6. 10.1359/jbmr.090533 19558316

[40] Fraser JRE, Laurent TC, Laurent UBG. Hyaluronan: its nature, distribution, functions and turnover. J Intern Med. 1997;242(1):27–33. 10.1046/j.1365-2796.1997.00170.x 9260563

[41] Balazs EA, Denlinger JL. Viscosupplementation: a new concept in the treatment of osteoarthritis. J Rheumatol Suppl. 1993;39:3–9. 8410881

[42] Petrey AC, de la Motte CA. Hyaluronan, a crucial regulator of inflammation. Front Immunol. 2014;5:101. 10.3389/fimmu.2014.00101 PMC3949149 24653726

[43] Campo GM, Avenoso A, Campo S, et al. Hyaluronic acid in part counteracts LPS-induced inflammation in mouse chondrocytes through TLR4 down-regulation. Int J ImmunoPathol Pharmacol. 2009;22(3):611–8.

[44] Ghosh P, Guidolin D. Potential mechanism of action of intra-articular hyaluronan therapy in osteoarthritis: are the effects molecular-weight dependent? Semin Arthritis Rheum. 2002;32(1):10–37. 10.1053/sarh.2002.33720 12219318

[45] Kim HA, Lee YJ. Septic arthritis and LPS-induced chondrocyte death. BMB Rep. 2013;46(11):543–8.

[46] Li Z, Wang Y, Xue Y, et al. Lipopolysaccharide induces mitochondrial dysfunction and apoptosis in chondrocytes via the TLR4/NF-κB pathway. Mol Med Rep. 2016;14(6):5539–46.

[47] Huang TL, Chen CH. Effects of hyaluronic acid on osteoarthritic chondrocytes: a review. Int J Rheum Dis. 2011;14(5):396–401.

[48] Hayden MS, Ghosh S. Regulation of NF-κB by TNF family cytokines. Semin Immunol. 2014;26(3):253–66. 10.1016/j.smim.2014.05.004 PMC4156877 24958609

[49] Oh JS, Kim YM, Song H, et al. High-molecular-weight hyaluronan suppresses TLR4-dependent inflammatory activation. J Biol Chem. 2010;285(29):21936–47.

[50] Campo GM, Avenoso A, Campo S, et al. Hyaluronic acid and chondroitin-4-sulfate inhibit TLR4 signalling by reducing NF-κB activation in human chondrocytes. Biochimie. 2010;92(10):1333–42.

[51] Aruffo A, Stamenkovic I, Melnick M, Underhill CB, Seed B. CD44 mediates hyaluronic-acid-dependent cell adhesion. Cell. 1990;61(7):1303–13. 10.1016/0092-8674(90)90694-a 1694723

[52] Hardingham TE, Bayliss MT. Proteoglycans of articular cartilage: changes in ageing and osteoarthritis. Semin Arthritis Rheum. 1990;20(3 Suppl 1):12–33. 10.1016/0049-0172(90)90044-g 2287945

[53] Roughley PJ, Mort JS. The role of aggrecan in normal and osteoarthritic cartilage. J Exp Orthop. 2014;1(1):8. 10.1186/s40634-014-0008-7 PMC4648834 26914753

[54] Kapoor M, Martel-Pelletier J, Lajeunesse D, Pelletier J-P, Fahmi H. Role of TNF-α and IL-1β in cartilage catabolism. Arthritis Res Therapy. 2010;12(1):207.

[55] Manderson AP, Kay JG, Hammond LA, Brown DL, Stow JL. Sub- compartments of the macrophage recycling endosome direct the differential secretion of IL-6 and TNF-α. J Cell Biol. 2007;178(1):57–69. 10.1083/jcb.200612131 PMC2064421 17606866

[56] Chen F, Liu Y, Wang H, et al. Lipopolysaccharide-induced cell-cycle arrest and apoptosis in chondrocytes via ROS-mediated pathways. Cell Stress Chaperones. 2015;20(4):667–75.

[57] Wu Y, Lu X, Chen Y, et al. Oxidative stress-induced G0/G1 arrest mediates chondrocyte apoptosis in osteoarthritis. Mol Med Rep. 2017;16(2):1658–64.

[58] Yang X, Guo L, Zhang Y, et al. Hyaluronic acid protects against oxidative DNA damage and apoptosis in chondrocytes. Exp Therapeutic Med. 2020;19(3):2091–8.

[59] Bowman S, Mikkelsen C, Petersen SG, et al. Intra-articular hyaluronic acid: clinical efficacy and molecular mechanisms. Clin Med Insights: Arthritis Musculoskelet Disorders. 2018;11:1–12.

[60] Bellamy N, Campbell J, Robinson V, Gee T, Bourne R, Wells G. Viscosupplementation for the treatment of osteoarthritis of the knee. Cochrane Database Syst Reviews. 2006;2:CD005321. 10.1002/14651858.CD005321.pub2 PMC8884110 16625635

[61] Migliore A, Provenzano G. Hyaluronic acid in the treatment of osteoarthritis: an update on molecular mechanisms and clinical evidence. Int J Rheumatol. 2019. 8980462.

[62] Stern R, Jedrzejas MJ. Hyaluronidases: their genomics, structures, and mechanisms of action. Chem Rev. 2006;106(3):818–39. 10.1021/cr050247k PMC2547145 16522010

[63] Lefebvre V, Dvir-Ginzberg M. SOX9 and the many facets of its regulation in the chondrocyte lineage. Connect Tissue Res. 2017;58(1):2–14. 10.1080/03008207.2016.1183667 PMC5287363 27128146

[64] Komori T. Regulation of proliferation, differentiation and functions of osteoblasts by RUNX2. Int J Mol Sci. 2019;20(7):1694. 10.3390/ijms20071694 PMC6480215 30987410

