# Supplementary material for: Effects of high-molecular-weight hyaluronic acid on inflammatory and matrix-associated responses in a chondrocyte–osteoclast co-culture model
Source: J Orthop Surg Res. 2026 Jul 13;21:580. doi: 10.1186/s13018-026-07024-7 (PMC13644255; doi:10.1186/s13018-026-07024-7)

Additional file 2. Supplementary data for Figure 3

Supplementary data related to Figure 3, including additional representative images and/or quantitative analysis of cellular metabolic activity and morphological observations in the chondrocyte–osteoclast co-culture system.

(A) MTT assay (24 h and 48 h)
Short description (optional):
Cellular metabolic activity under control, LPS, and HA-treated conditions.

(B) Morphological observation – Control
Bright-field image showing normal cell morphology.

Control

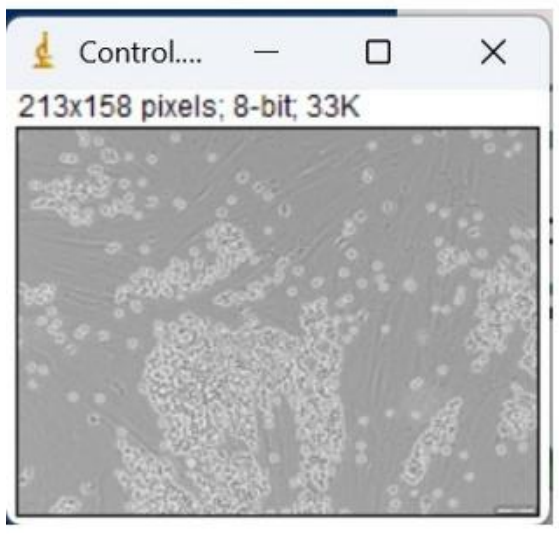

Threshold

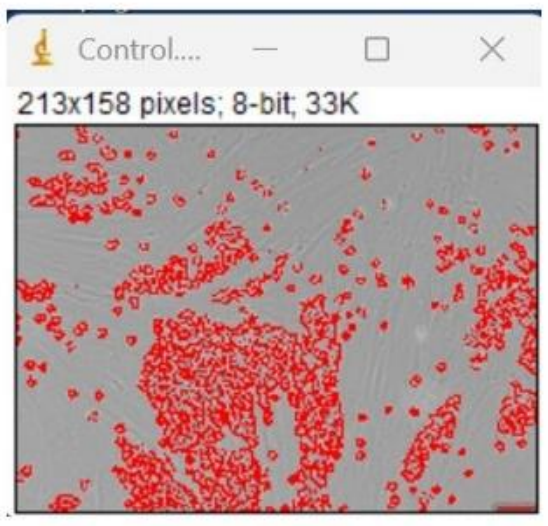

C Binary mask

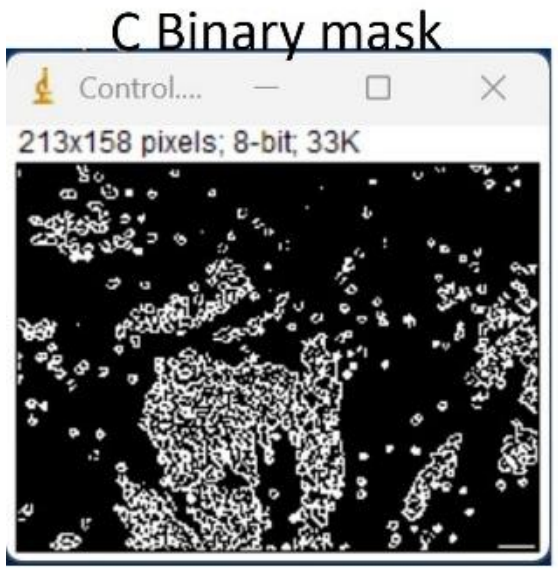

Outlines and Results

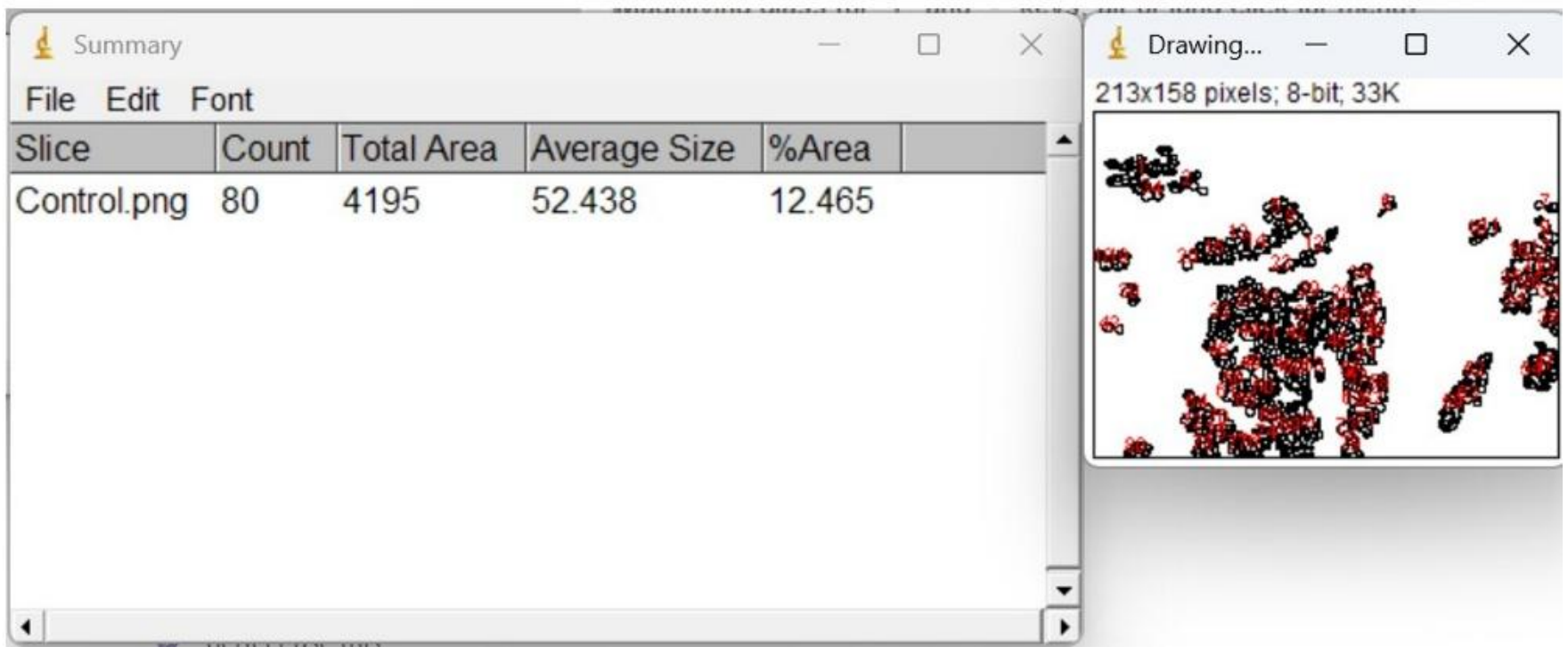

Slice	Count	Total Area	Average Size	%Area
Control.png	80	4195	52.438	12.465

(C) Morphological observation – LPS-treated

Cells showing reduced spreading and structural disruption

Control

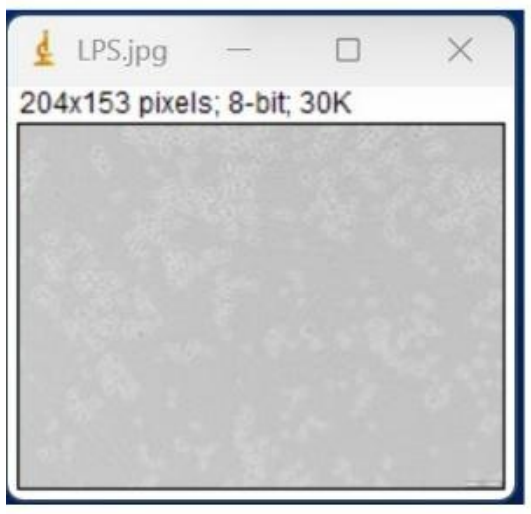

Threshold

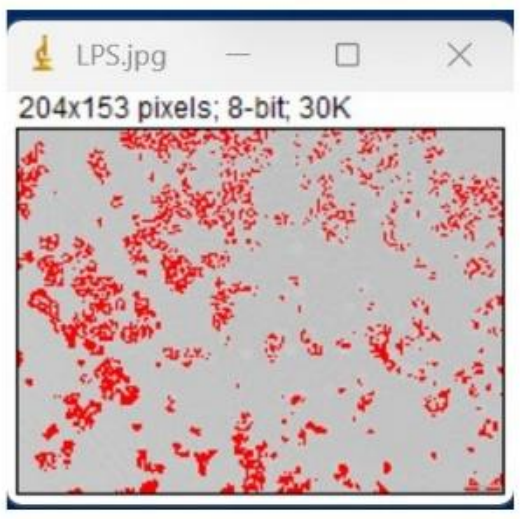

Binary mask

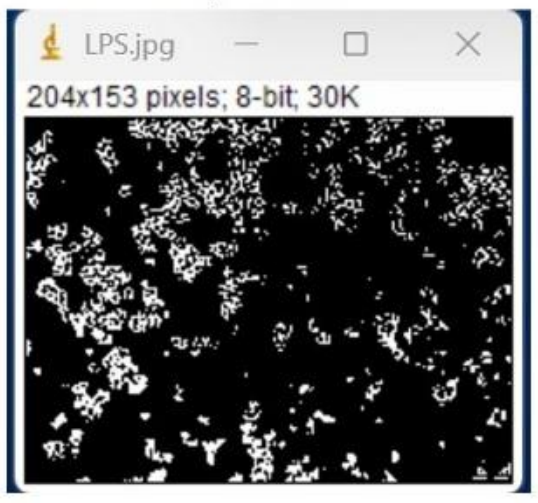

Outlines and Results

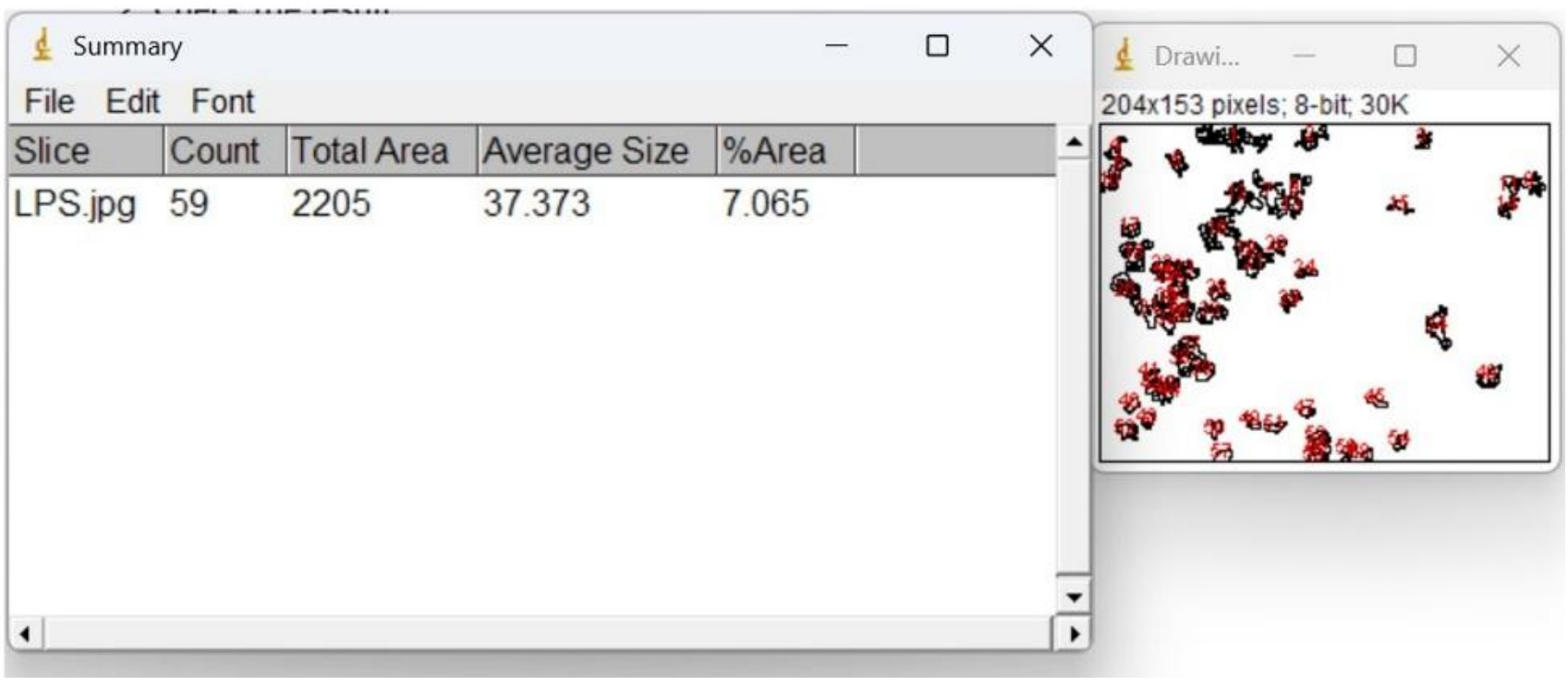

(D) Morphological observation – HA-treated

Partial restoration of morphology following HA treatment.

Control

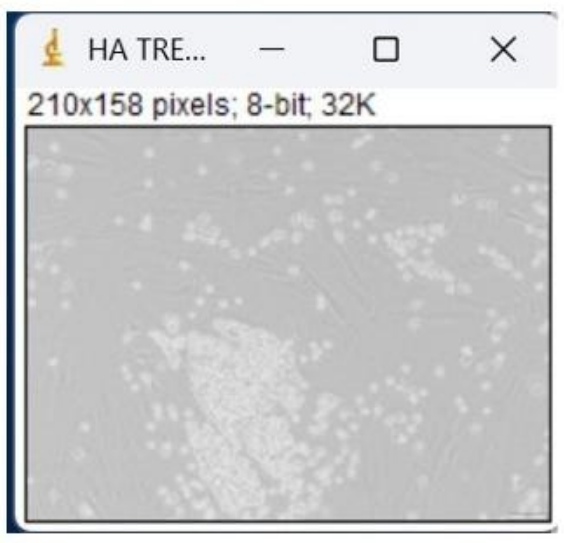

Threshold

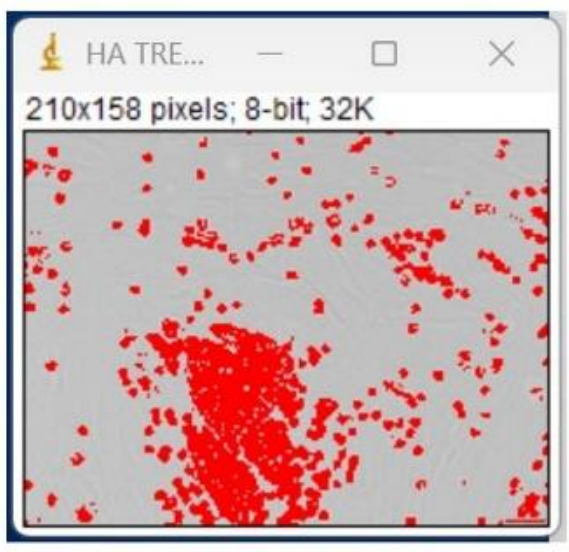

Binary mask

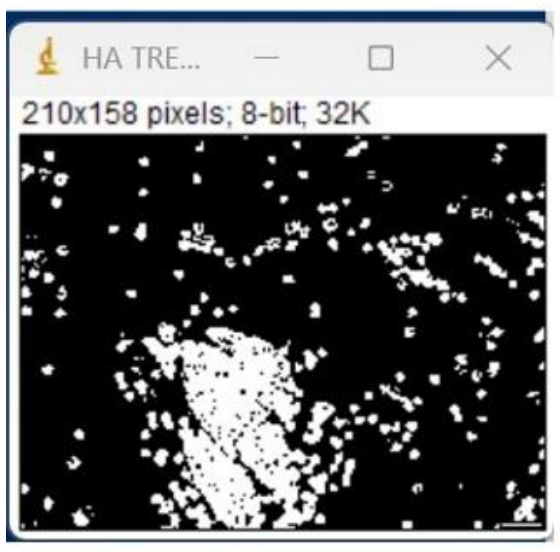

Outlines and Results

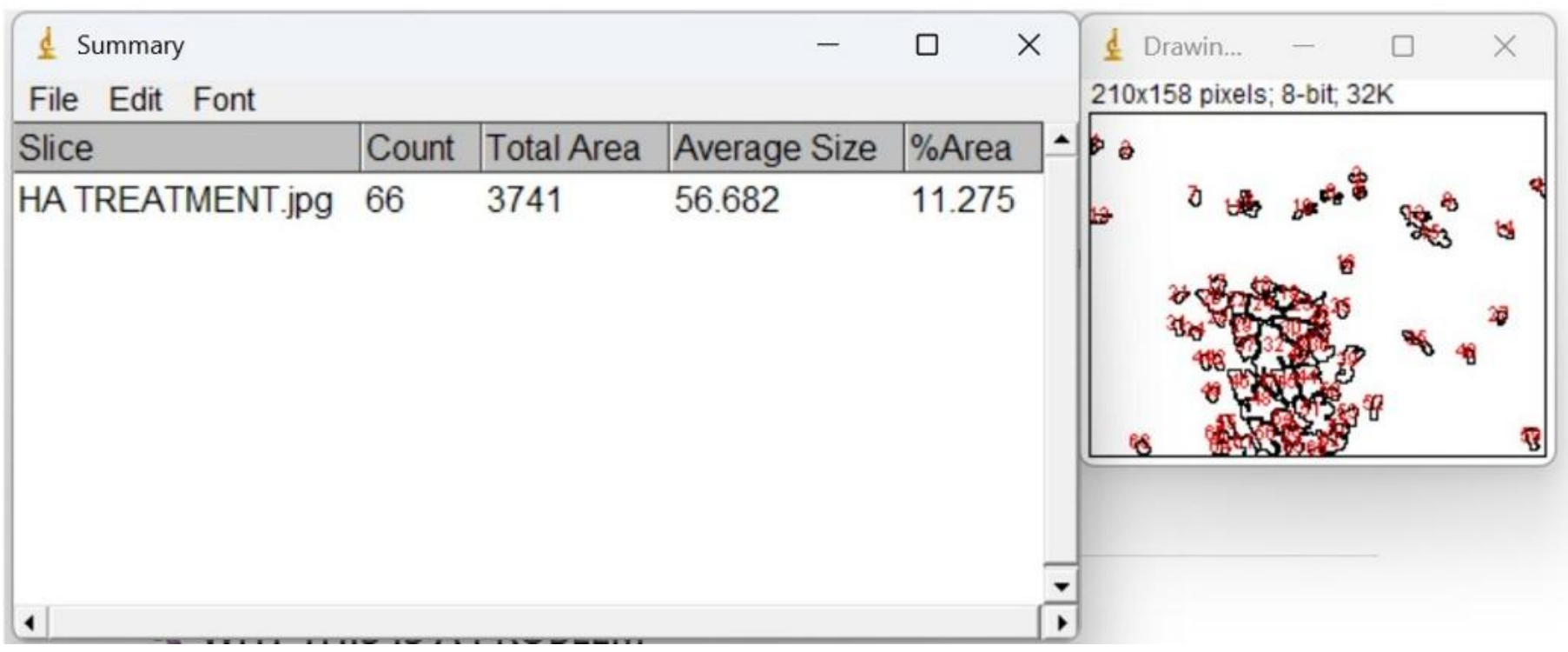

Supplement: Supplementary file 1 [file 13018_2026_7024_MOESM1_ESM.pdf]
