# Supplementary material for: Effects of high-molecular-weight hyaluronic acid on inflammatory and matrix-associated responses in a chondrocyte–osteoclast co-culture model
Source: J Orthop Surg Res. 2026 Jul 13;21:580. doi: 10.1186/s13018-026-07024-7 (PMC13644255; doi:10.1186/s13018-026-07024-7)
Supplement: Supplementary file 2 [file 13018_2026_7024_MOESM2_ESM.pdf]

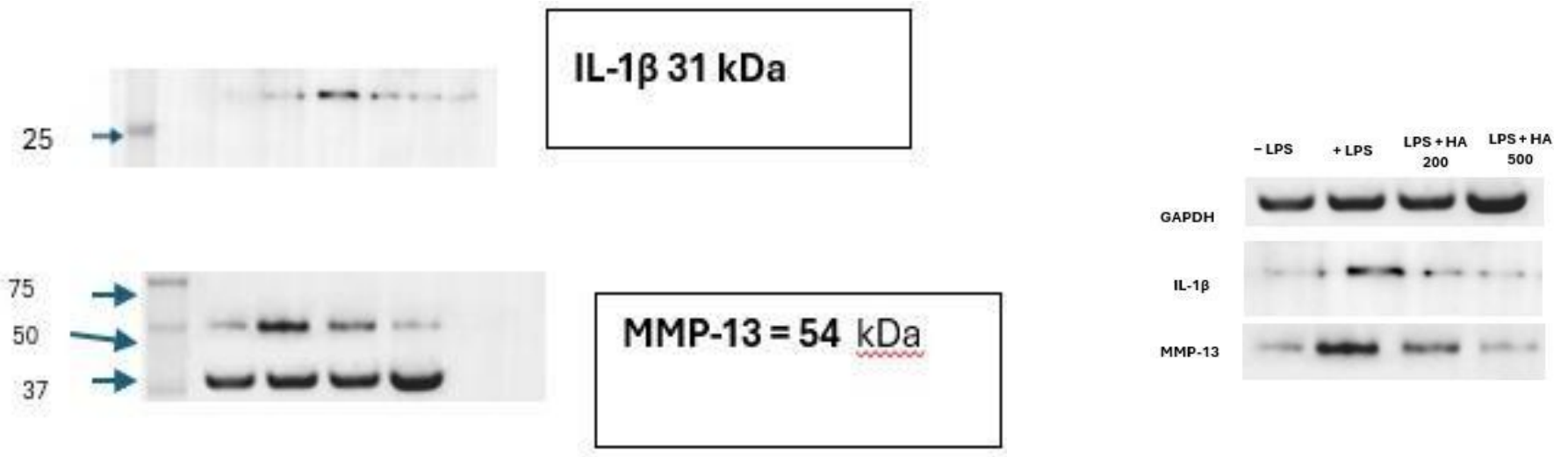

Supplementary Figure S1A.

Available original Western blot membrane regions corresponding to IL-1β (~31 kDa) and MMP-13 (~54 kDa).

The images shown represent the original captured membrane regions from the same membrane, including molecular weight markers where available. The cropped bands used for densitometric analysis in the main manuscript were derived directly from these original membrane regions.

Membranes were sectioned based on molecular weight prior to antibody incubation and processed for specific targets; therefore, full-length membranes are not presented as a single continuous image. All available original membrane regions corresponding to the target proteins are provided, including molecular weight markers where available.

All samples were loaded in the same lane order across all membrane regions.

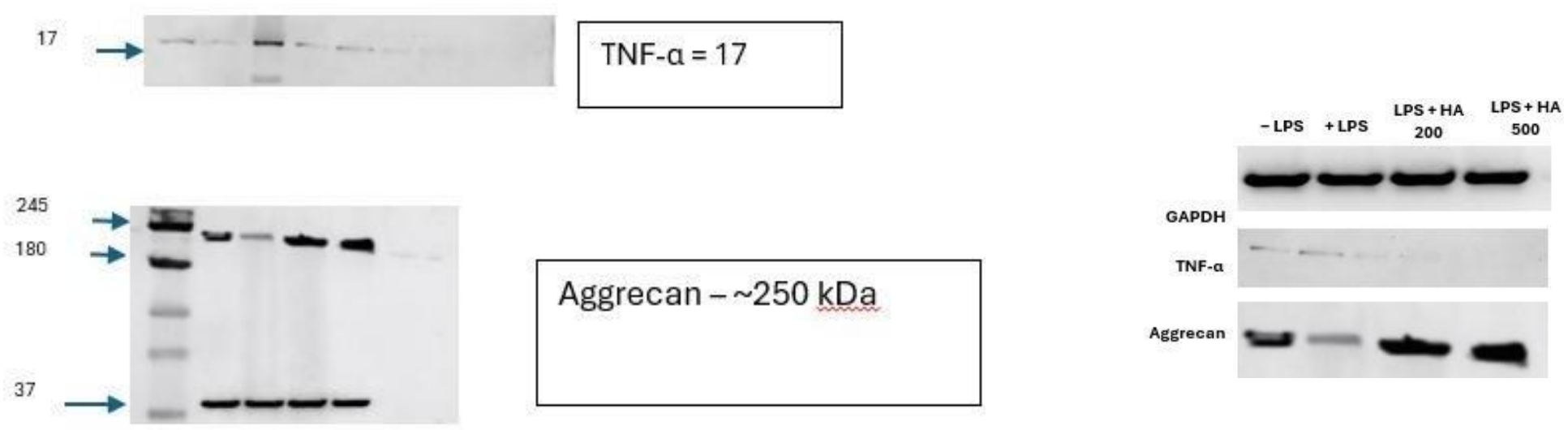

Supplementary Figure S1B.

Available original Western blot membrane regions corresponding to TNF-α (~17 kDa) and Aggrecan (~250 kDa).

The images shown represent the original captured membrane regions from the same membrane, including molecular weight markers where available. The cropped bands used for densitometric analysis in the main manuscript were derived directly from these original membrane regions.

Membranes were sectioned based on molecular weight prior to antibody incubation and processed for specific targets; therefore, full-length membranes are not presented as a single continuous image. All available original membrane regions corresponding to the target proteins are provided, including molecular weight markers where available.

All samples were loaded in the same lane order across all membrane regions.

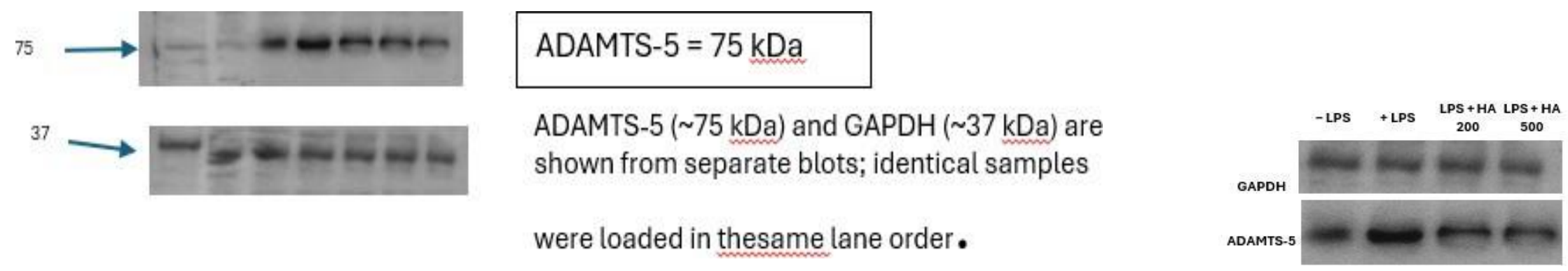

Supplementary Figure S1C.

Available original Western blot membrane regions corresponding to ADAMTS-5 (~75 kDa) and GAPDH (~37 kDa).

The images shown represent the original captured membrane regions used for protein detection, including molecular weight markers where available. ADAMTS-5 and GAPDH are presented from separate blots, as additional target proteins were analysed between these molecular weight ranges, requiring independent membrane processing.

The cropped bands used for densitometric analysis in the main manuscript were derived directly from these original membrane regions.

All samples were run under identical experimental conditions and loaded in the same lane order across blots to ensure consistency and enable valid comparison.

Membranes were sectioned based on molecular weight prior to antibody incubation and processed separately; therefore, full-length membranes are not presented as a single continuous image. All available original membrane regions are provided.

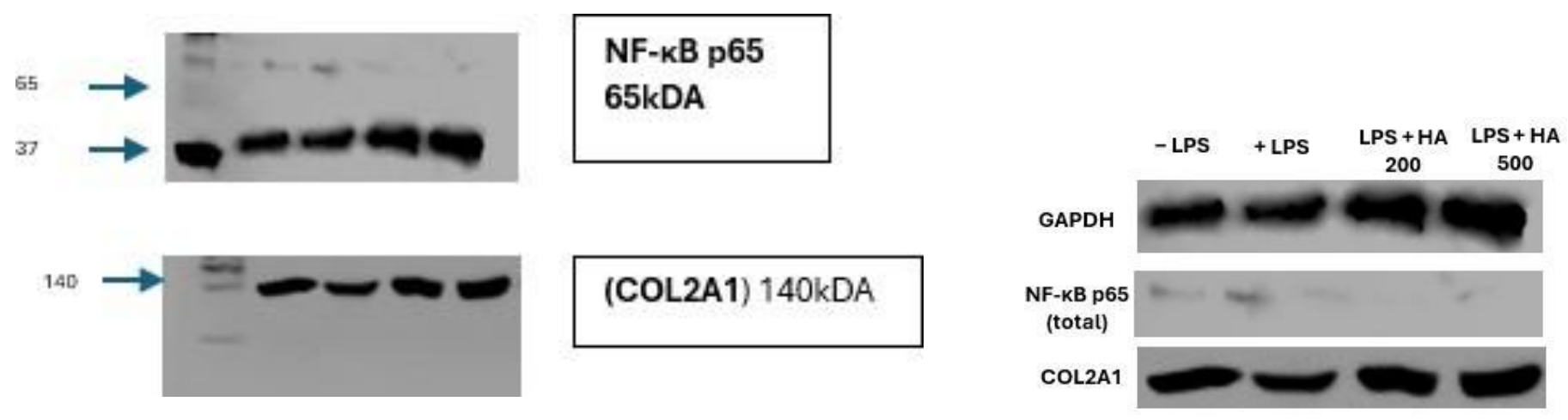

Supplementary Figure S1D.

Available original Western blot membrane regions corresponding to NF-κB p65 (~65 kDa) and COL2A1 (~140 kDa).

The images shown represent the original captured membrane regions from the same membrane, including molecular weight markers where available. The cropped bands used for densitometric analysis in Figure 4D of the main manuscript are presented alongside the corresponding GAPDH loading control.

The cropped bands used for densitometric analysis in the main manuscript were derived directly from these original membrane regions.

Membranes were sectioned based on molecular weight prior to antibody incubation and processed for specific targets; therefore, full-length membranes are not presented as a single continuous image. All available original membrane regions corresponding to the target proteins are provided, including molecular weight markers where available.

All samples were loaded in the same lane order across all membrane regions**.**
